# The importance of regulated resource reallocation during dynamic environmental shifts in yeast

**DOI:** 10.1038/s44318-026-00727-x

**Published:** 2026-03-11

**Authors:** Rachel A Kocik, Eli G Cytrynbaum, Jamie M Ahrens, Megan N McClean, Audrey P Gasch

**Affiliations:** 1 Center for Genomic Science Innovation, University of Wisconsin-Madison, Madison, WI 53706 USA; 2 Department of Biomedical Engineering, University of Wisconsin-Madison, Madison, WI 53706 USA; 3 Department of Medical Genetics, University of Wisconsin-Madison, Madison, WI 53706 USA

**Keywords:** Stress, Resource Allocation, Regulatory Dynamics, Chromatin, Transcription & Genomics, Evolution & Ecology

## Abstract

Many organisms maintain generalized stress responses activated by adverse conditions. A common theme is the induction of stress-defense proteins with reduced production of growth-promoting proteins, including ribosomes. Yet the precise roles of these coupled programs are difficult to dissect. Here, we investigated *Saccharomyces cerevisiae* responding to salt as a model stressor. We used molecular, genomic, and single-cell microfluidic methods to examine the interplay between transient induction of stress-defense genes and coordinated repression of growth-promoting genes in the yeast environmental stress response (ESR). Loss of transcriptional inducers Msn2/4 accelerates growth during multiple mild stress doses, at the expense of acquired tolerance to subsequent severe stresses. In contrast, loss of Dot6/Tod6 repressors of growth-promoting genes delays stress acclimation, showing that gene repression accommodates the cost of the Msn2/4 response. Msn2/4 bind the *DOT6* promoter, influence Dot6 abundance and activation dynamics, and are required for full repression of Dot6 targets and other growth-promoting genes. Thus, Msn2/4 participate in regulating resource reallocation needed to induce their transcripts, underscoring a common theme in stress responses utilized in other organisms.

## Synopsis

How do genes induced and repressed in the yeast environmental stress response (ESR) contribute to stress tolerance, growth rate, and resource allocation, during single- versus multi-stress exposures? This work uses molecular, genomic, and systems biology approaches to present new insights into eukaryotic responses to acute stress.

- Cells lacking stress-activated transcription factors have a faster post-stress growth rate.
- Loss of stress-activated gene repression produces a delay in acquired stress tolerance.
- Transcriptional activators of defense genes contribute to gene repression to coordinate resource re-allocation needed for the response.

## Abstract (web summary)

Molecular, genomic and systems-biology approaches reveal how genes induced or repressed in the environmental stress response contribute to stress tolerance and growth rate.

## Graphical abstract

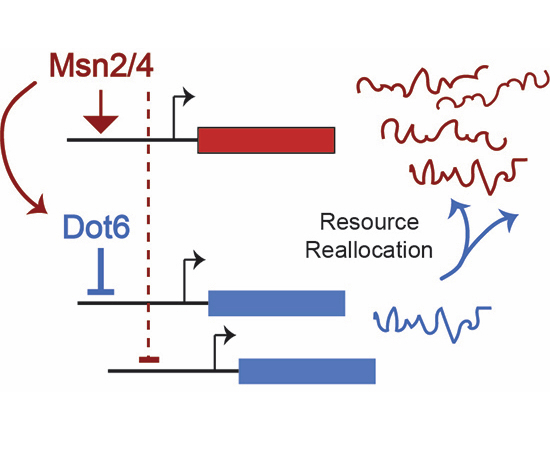

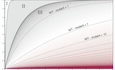

## Introduction

Cells have evolved intricate systems to allocate limited intracellular resources according to the demands of an often-fluctuating environment. When conditions are favorable, many microbes maximize their growth rate by directing resources toward growth and proliferation. Much of the transcriptional and translational capacity goes toward producing ribosomes, which under optimal conditions fuel rapid growth (Scott and Hwa, 2011, 2023; Warner, 1999). However, in suboptimal conditions, especially in response to acute stress, resources including transcriptional and translational capacity are reallocated toward survival, often at the expense of growth and growth-promoting processes. In fact, rapid growth and high stress tolerance represent a well-known tradeoff: fast-growing cells directing resources toward division are often the most sensitive to stress, whereas slow growing cells are typically highly stress tolerant (Balaban et al, 2004; Basu et al, 2022; Levy et al, 2012; Pontes and Groisman, 2019; Zakrzewska et al, 2011; Zhang et al, 2020). This is true across organisms, including bacteria, yeast, plants, and mammalian cells. However, it remains poorly understood how cells regulate changes in resource allocation during times of stress and which cellular objectives (maximizing growth versus high stress tolerance) dictate those changes. This is important for understanding how cells thrive in natural environments that are dynamic and often suboptimal. Presumably, cells must coordinate multiple facets of physiology as they respond and acclimate to changing conditions.

**Figure 1:**
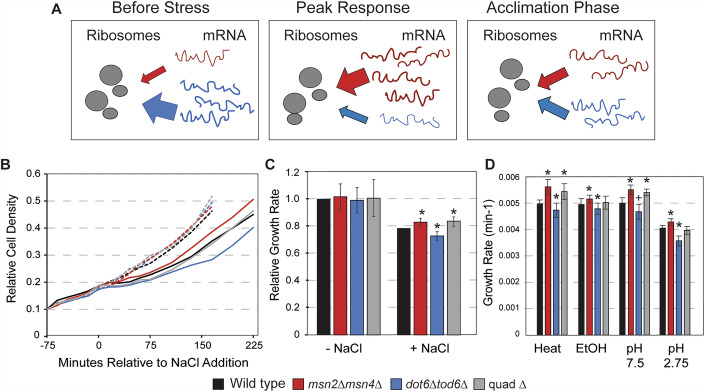
Msn2/4 versus Dot6/Tod6 have opposing influences on post-stress growth rate. (**A**) A model for the dynamics of stress-defense mRNAs induced by stress (red) versus growth-promoting mRNAs highly expressed in optimal conditions but transiently repressed during stress acclimation (blue). (**B**) Representative relative cell density (OD_600_) for wild-type (black), *msn2∆msn4∆* (red), *dot6∆tod6∆* (blue), and quadruple mutant (“quad∆”) (gray) growing in the absence (dashed lines) and presence (solid lines) of 0.7 M NaCl added at 0 min. (**C**) Average and standard deviation (*n* = 7) of growth rates in the absence (left) and presence (right) of 0.7 M NaCl (calculated from 75 to 225 min timepoints). Post NaCl growth rates were calculated relative to their paired wild-type, then scaled to the average wild-type post-stress versus pre-stress relative rate. Exact *P* values from replicate-paired, two-sided *T* tests are listed in Dataset EV1. (**D**) Average and standard deviation (*n* = 6) of growth rates after 30–40 °C heat shock, 3% ethanol, pH 7.5 or 2.5 shift. *P value < 0.01, +*P* value = 0.07, two-tailed, replicate-paired *t* test relative to the corresponding treated wild-type. Exact *P* values are listed in Dataset EV1. Source data are available online for this figure.

Budding yeast *Saccharomyces cerevisiae* has been an excellent model to understand principles of growth-versus-defense responses. Upon an acute shift to suboptimal conditions, yeast activate condition-specific responses customized for each condition, along with a common transcriptomic response known as the environmental stress response (ESR) (Causton et al, 2001; Gasch et al, 2000). The ESR is activated in response to diverse types of stress, including nutrient limitation, shifts in environmental conditions like osmolarity or temperature, and exposure to toxic compounds. The program includes ~300 transcriptionally induced genes (iESR genes) involved in wide-ranging defense processes such as redox balance, protein folding and degradation, carbohydrate and energy metabolism, trehalose and glycogen biosynthesis, and other processes (Gasch, 2002a; Gasch et al, 2000). Induced transcription of iESR genes is coordinated with reduced expression of ~600 genes (rESR genes) that encode ribosomal proteins (RP) and proteins involved in ribosome biogenesis, translation, and other growth-promoting processes (RiBi genes) (Gasch, 2002a; Gasch et al, 2000). In optimal conditions, cells devote significant resources to transcribing and translating rESR transcripts, which are required for rapid growth, while maintaining low production of iESR and defense proteins (Fig. 1A). Notably, other organisms maintain analogous, if not orthologous, responses to balance stress-defense versus growth-promoting processes, including the Integrated Stress Response (ISR) in mammals (Costa-Mattioli and Walter, 2020; Harding et al, 2003) and the Stringent/SOS responses in bacteria (Gourse et al, 2018; Irving et al, 2021). Despite differences in the regulation of these programs across species, many of the underlying themes are conserved, including the redirection of cellular resources away from growth-promoting processes and toward stress-induced transcripts. Yet decoupling the role of translational suppression from the functions of induced proteins has remained challenging.

iESR induction and rESR repression are highly correlated in bulk cultures, at least in part due to coordinated regulation (Gasch, 2002a). iESR gene induction is partially orchestrated by the paralogous “general stress” transcription factors Msn2 and Msn4 (Causton et al, 2001; Estruch and Carlson, 1993; Gasch et al, 2000), overlaid with condition-specific factors that customize expression levels in specific environments (Gasch, 2002a). Msn2 and/or Msn4 (Msn2/4) bind the stress response promoter element (STRE, CCCCT) present in one to many copies upstream of hundreds of target genes (Martínez-Pastor et al, 1996; Stewart-Ornstein et al, 2013). Like many stress-regulated factors, Msn2/4 are regulated by nuclear translocation: phosphorylation at specific residues, including by growth-promoting Protein Kinase A (PKA) and TOR kinases, restricts the factors to the cytoplasm during optimal conditions (Beck and Hall, 1999; Boy-Marcotte et al, 1998; Görner et al, 1998; Jacquet et al, 2003; Smith et al, 1998). During stress, dephosphorylation of those residues coupled with other activating mechanisms prompts Msn2/4 nuclear localization and gene induction (De Wever et al, 2005; Garreau et al, 2000; González et al, 2009; Görner et al, 1998; Lenssen et al, 2005; Santhanam et al, 2004; Smith et al, 1998). The dynamics of nuclear translocation can impart important differences in gene expression, depending on the amount of protein that enters the nucleus, the duration of nuclear accumulation, and gene-promoter architecture including the number of upstream STRE elements of each gene (Gasch, 2002a; Hansen and O’Shea, 2013, 2015a, 2015b, 2016; Hansen and Zechner, 2021; Hao and O’Shea, 2012; Purvis et al, 2012; Purvis and Lahav, 2013; Stewart-Ornstein et al, 2013; Sweeney and McClean, 2023). rESR subgroups are modulated by different regulators (Gasch, 2002a; Jorgensen et al, 2004; Marion et al, 2004; Schawalder et al, 2004; Shore and Nasmyth, 1987). Many of the RiBi genes are repressed during stress by Dot6 and its paralog Tod6, each of which can dominate the response depending on the conditions (Bergenholm et al, 2018; Cheng and Brar, 2019; Lippman and Broach, 2009). Dot6 and/or Tod6 (Dot6/Tod6) bind the GATGAG motif, which is present in about ~70% of RiBi gene promoters (Badis et al, 2008; Zhu et al, 2009). Furthermore, Dot6/Tod6 are also regulated by nuclear translocation, where phosphorylation by PKA and Sch9/TOR maintains the factors in the cytoplasm and dephosphorylation leads to nuclear accumulation (Huber et al, 2011; Lippman and Broach, 2009).

During many stress responses, ESR activation, and in particular rESR repression, coincides with growth reduction; however, the ESR is not an indirect response to growth as previously proposed (see (Brauer et al, 2008; Castrillo et al, 2007; Lee et al, 2011; Lu et al, 2009; O’Duibhir et al, 2014; Regenberg et al, 2006)). Cells already arrested in division and with reduced biomass production still show rESR repression upon stress exposure (Ho et al, 2018). We proposed that rESR repression during acute stress helps to redirect transcriptional and translational capacity toward induced mRNAs (Bergen et al, 2022; Ho et al, 2018; Lee et al, 2011). Cells lacking *DOT6/TOD6* fail to fully repress rESR genes during acute salt stress, leading to the over-abundance of rESR transcripts that remain associated with ribosomes. In turn, iESR transcripts show reduced ribosome binding and delayed production of their proteins (Ho et al, 2018; Lee et al, 2011). Indeed, the *dot6∆tod6∆* mutant also shows delayed synthesis of iESR protein Ctt1 encoding cytosolic catalase, despite higher induction of *CTT1* mRNA (Bergen et al, 2022; Ho et al, 2018). Thus, repression of the rESR genes may indirectly influence the production of induced proteins simply by decreasing rESR mRNAs from the translating pool.

Studying cell cultures in bulk can obscure causal relationships that vary across individual cells in a population. Thus, investigating cell-to-cell heterogeneity has been a useful tool in deciphering co-varying phenotypes that can reflect on cellular coordination (Bagamery et al, 2020; Barber et al, 2021; Gasch et al, 2017; Levy et al, 2012; Li et al, 2018). We previously used microfluidic live-cell imaging to study single-cell heterogeneity in cells responding to an acute dose of sodium chloride (NaCl) stress (Bergen et al, 2022). Our system enabled characterization of multiple phenotypes in single cells, including growth rate, colony size, cell-cycle phase, and nuclear-translocation dynamics of fluorescently tagged Msn2-mCherry and Dot6-GFP expressed in the same cells. Somewhat counterintuitively, we found that wild-type cells with larger Dot6 nuclear translocation response, predicted to produce stronger repression of growth-promoting rESR genes, in fact acclimate with faster post-stress growth rates following acute salt stress. Wild-type cells with stronger Dot6 activation also displayed faster production of Ctt1 protein compared to cells with weaker activation, consistent with our model that rESR repression helps to accelerate production of induced proteins (Bergen et al, 2022). We proposed that Dot6-dependent transcriptional repression, and by extension repression of the rESR as a whole, is important to reallocate resources for faster acclimation to stress conditions. But if and how a faster response is important for stress survival has not been tested. Furthermore, how resource reallocation through gene repression is coordinated with genes induced in the ESR was not clear from past work.

Here, we investigated the interplay between Msn2/4 and Dot6/Tod6 activation dynamics, transcriptional regulation, and growth versus defense objectives. We show that activation of the Msn2/4 response comes at a significant cost during a single-stress treatment, in a manner that is accommodated by Dot6/Tod6-dependent repression. Yet both programs are required for normal acquisition of subsequent stress tolerance when cells are shifted between two stressful conditions. By analyzing our own and previously published datasets, we show that Msn2/4 contribute to the regulation of Dot6 and many other genes repressed in the rESR, demonstrating that Msn2/4 help manage the resource reallocation needed for their own response. We discuss reminiscent programs in the stress responses of other species, even when the precise mechanisms have evolved.

## Results

### Msn2/4 versus Dot6/Tod6 have opposing influences on growth rate after a single-stress treatment

We set out to test the separable effects of iESR and rESR expression changes by studying cells lacking their respective regulators. We previously showed that cells lacking Dot6 and Tod6 (*dot6∆tod6∆)* grow indistinguishably from wild-type in the absence of stress but display slower growth after salt stress (Bergen et al, 2022). We confirmed here that *dot6∆tod6∆* cells show a similar lag phase but reduced growth rate compared to wild-type cells only after stress treatment (*P* = 0.002, replicate-paired *T* test (Fig. 1B,C). The reduced post-stress growth rate was also seen for other stresses, including heat shock, ethanol, basic or acid pH treatments (Fig. 1D), and was previously reported after nitrogen and glucose deprivation (Lippman and Broach, 2009). A remaining question was the role of Msn2/4 and iESR induction on stress acclimation. Like the *dot6∆tod6∆* mutant, *msn2∆msn4∆* cells grew indistinguishably from wild-type in the absence of stress (Fig. 1B). Surprisingly, however, the *msn2∆msn4∆* mutant grew significantly faster than wild-type cells after salt stress (*P* = 0.008) and with shorter lag, since they had a significantly greater percent change in cell density over 60 min compared to the wild-type (*P* = 0.010, replicate and time-paired two-tailed *T* test). The growth rate trend was reproducible across other stresses as well (Fig. 1D). Thus, mounting the iESR comes with a significant cost to post-stress growth rate after a single-stress treatment.

We reasoned that if Dot6/Tod6-dependent repression serves to release resources for induced protein production, then loss of the costly Msn2/4 response may recover growth rate in *dot6∆tod6∆* cells. Alternatively, if Dot6/Tod6 plays a different unrecognized role, deletion of *MSN2/4* would not alleviate its post-stress growth requirement. To test this, we generated a strain lacking all four transcription factors (referred to as the quad mutant or *quad∆*). Like both double mutants, the quad mutant grew similarly to wild-type in the absence of stress. However, during 75–225 min post-salt addition, the quad mutant grew faster than the wild-type, similar to cells lacking Msn2/4. The result held for other stresses as well, where the quad mutant recovered with post-stress growth that was equal to or better than the wild-type strain. Thus, the reduced post-stress growth rate of *dot6∆tod6∆* cells acclimating to stress can be complemented by loss of Msn2/4, suggesting that the cost of iESR induction explains the fitness defect when RiBi gene repression is lost.

### Both Msn2/4 and Dot6/Tod6 responses benefit future-stress survival

**Figure 2:**
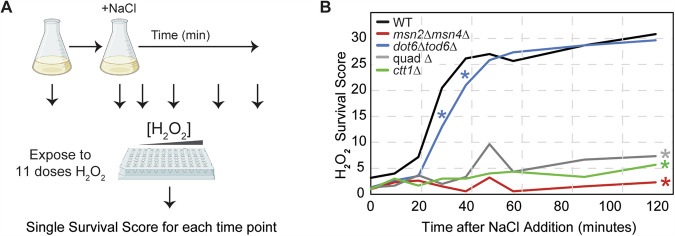
Msn2/4 and Dot6/Tod6 responses are important for acquired stress resistance. (**A**) Schematic of acquired stress resistance protocol, see text. (**B**) The average change in H_2_O_2_ survival scores for wild-type (black), *msn2∆msn4∆* (red), *dot6∆tod6∆* (blue), quad ∆ (gray), and *ctt1*∆ (green) cells. *n* = 3 replicates except for *dot6∆tod6∆* where *n* = 6. *P value < 0.03 (*), one-tailed, replicate-paired *t* test at each timepoint (see Fig. EV1 for paired datasets used in statistics). *P* values for *dot6∆tod6∆* versus WT: 0.030 (30 min), 0.0186 (40 min). The *msn2∆msn4∆, quad∆,* and *ctt1∆* mutants were highly significant at all time points after 20 min (represented by a single asterisk at the end of the curve, see Dataset EV3 for exact *P* values). Source data are available online for this figure.

**Figure EV1:**
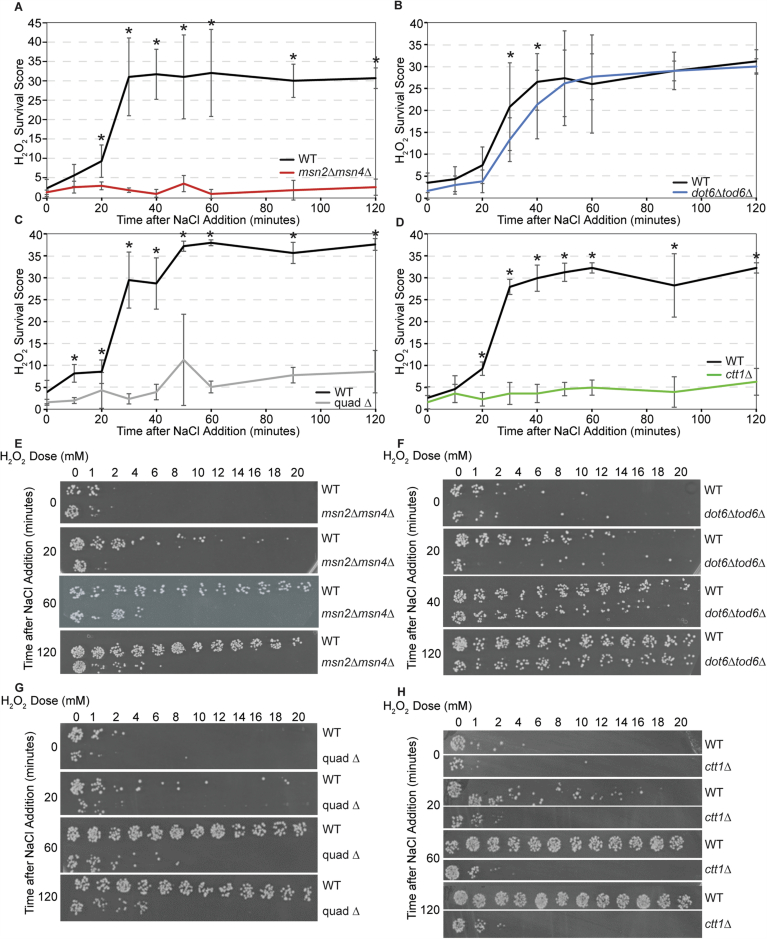
Msn2/4 and Dot6/Tod6 responses are important for acquired stress resistance. Related to Fig. 2. (**A**–**D**) The average change in H_2_O_2_ survival scores for wild-type (black), *msn2∆msn4∆* (red), *dot6∆tod6∆* (blue), quad ∆ (gray), and *ctt1*∆ (green) cells +/− 1 standard deviation, as described in Fig. 2 (*n* = 3 except for (**C**) where *n* = 6). Colored lines are as shown in Fig. 2, along with the paired wild-type culture done side-by-side with each mutant. (**E**–**H**) Representative images of cell viability across doses of H_2_O_2_ and time used to calculate H_2_O_2_ survival scores. Paired *t* test, exact *P* values are available in Dataset EV1. Source data are available online for this figure.

The cost of Msn2/4 activity to post-stress growth rate raised questions about why cells would maintain this response. One explanation is acquired stress resistance, in which cells that mount a stress response during a mild dose of one stress can survive what would otherwise be a lethal dose of subsequent stress treatment (Berry and Gasch, 2008). Past work from our lab showed that Msn2/4 are essential for acquired resistance to severe peroxide stress after salt-stress pretreatment (Berry et al, 2011; Berry and Gasch, 2008), which we confirmed here. At varying times before and after NaCl treatment, an aliquot of culture was removed and cells were exposed to a panel of H_2_O_2_ doses for 2 h, after which colony forming units were assessed (Fig. 2A). The relative viability at each dose was normalized to the side-by-side treated wild-type, and a single H_2_O_2_ survival score was calculated as the sum of those scores across doses (see “Methods”, Fig EV1A–H). As expected, cells lacking *MSN2/4* had a major defect acquiring peroxide tolerance after salt treatment, as did the quad mutant that also lacks *MSN2/4* (Fig. 2B). As shown previously, acquisition of peroxide tolerance was also dependent on Msn2/4 target *CTT1* encoding cytosolic catalase, since a strain lacking *CTT1* acquired little tolerance (Berry and Gasch, 2008; Guan et al, 2012) (Fig. 2B). Thus, the Msn2/4 response, at least in part via Ctt1, is essential for acquired peroxide tolerance under these conditions.

The role of Dot6/Tod6 and rESR repression in acquired stress resistance had not been previously investigated. Here, we found that Dot6/Tod6 are also required for normal acquisition of peroxide treatment. While the *dot6∆tod6∆* mutant acquired wild-type levels of hydrogen peroxide resistance after exposure to salt, they did so with a significant delay (Fig. 2B). Wild-type cells acquired maximal resistance by ~40 min; however, the *dot6∆tod6∆* cells took over ~60 min to reach maximal tolerance. This delayed acquisition of peroxide tolerance cannot be explained by the reduced growth rate of the mutant, which was observed at later time points (see Fig. 1). Instead, the timing of the delay correlates with delayed Ctt1 protein production in the *dot6∆tod6∆* mutant (Bergen et al, 2022; Ho et al, 2018). These results are consistent with the model that Dot6/Tod6 gene repression helps to accelerate production of stress-defense proteins needed for acquired stress resistance.

### Interplay between Msn2 and Dot6 activation dynamics

While response couplings are often difficult to identify in bulk cultures, co-varying phenotypes become apparent when scoring single-cell heterogeneity within a population. We previously developed a microfluidics assay to explore heterogeneity in the nuclear translocation dynamics of Msn2-mCherry and Dot6-GFP expressed in the same cells (Bergen et al, 2022). In that study, we found that wild-type cells with a larger peak in Dot6 nuclear accumulation acclimated with faster post-stress growth rates than cells with a smaller peak. Here, we characterized how each ESR response impacted activation of the counterpart ESR regulators.

**Figure EV2:**
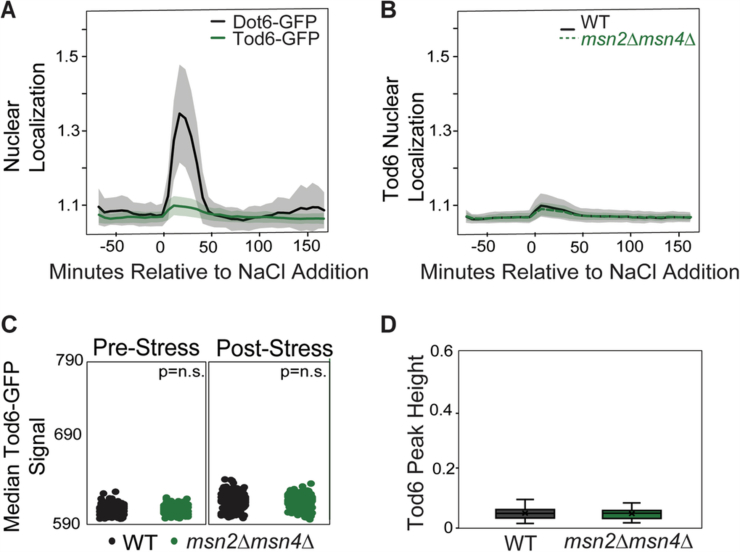
Loss of *MSN2/4* leads does not affect Tod6 abundance and nuclear localization. *Y*-axes are set to scales comparable to Fig. 3B–D for comparison with Dot6. (**A**) Dot6-GFP in an iRFP-marked strain was mixed with cells expressing Tod6-GFP, both expressed from the native genomic loci, and nuclear translocation of each factor was scored before and after NaCl treatment in the same device (*n* = 132 cells: WT, 56, *msn2∆msn4∆*, 76). The results confirm that Dot6-GFP shows strong nuclear relocalization in response to NaCl, whereas Tod6-GFP shows only a weak change under the conditions used here. (**B**) The population average of Tod6-GFP nuclear/cytoplasmic ratio in wild-type (black line) and *msn2∆msn4∆* (dashed green line) cells +/− one standard deviation (shading). The two strains are statistically indistinguishable (*P* > 0.1) (**C**) Distribution of median Tod6-GFP signal scored before (0–72 min) or after (120–216 min) NaCl treatment (see “Methods” for details) for WT and *msn2∆msn4∆* cells; *P* = 0.138 (left panel), 0.817 (right panel), Wilcoxon rank-sum test; n.s. = not significant. (**D**) Distribution of Tod6-GFP acute-stress peak heights across 132 cells, see “Methods”. *P* = 0.896, Wilcoxon rank-sum test. Boxplots show median (line) and 0.25 and 0.75 quartiles (box), with whiskers extending from minimum to maximum excluding outliers (circles) that are <0.25 quartile–1.5× interquartile range or >0.75 quartile–1.5× interquartile range. This figure shows that cells lacking *MSN2/MSN4* show no difference in Tod6-GFP levels or activation compared to wild-type. Source data are available online for this figure.

**Figure EV3:**
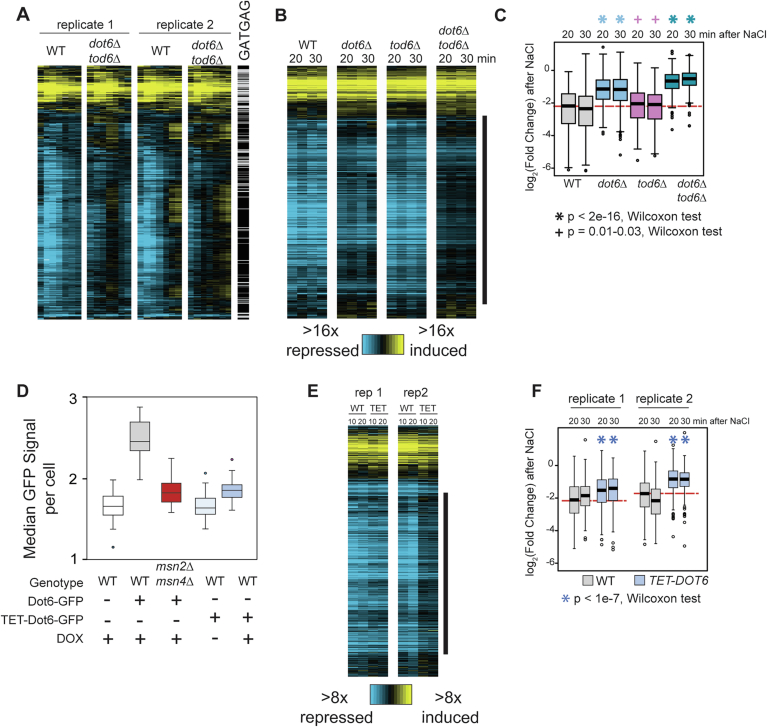
Dot6 contributes to gene repression more than Tod6 during NaCl stress. (**A**) Log_2_(fold change) of 489 genes significantly affected by *dot6∆tod6∆* deletion in the time course (10–90 min), as shown in Fig. 5A, see Methods for details. Presence of GATGAG sequences within 500 bp upstream of each gene is indicated with a black line next to each gene. (**B**) Expression of the same genes in single-gene deletion of *DOT6* or *TOD6* at the peak of the expression response (20 and 30 min). (**C**) Boxplots of replicate-averaged data from the highlighted cluster from (**B**) indicated with the black bar (379 genes). The median repression of wild-type at 20 min is indicated with a red-dashed line. Note the *dot6∆* mutant has a partial repression defect that is highly statistically significant compared to the wild-type (*P* < 2e-16, Wilcoxon rank-sum test), whereas the *tod6∆* mutant shows only subtle repression differences compared to the wild-type with marginal significance (*P* = 0.01–0.03, Wilcoxon rank-sum test). (**D**) The distribution of median Dot6-GFP levels in individual cells of indicated strains (*n* = 50 each strain) exposed to 50 µg/mL doxycycline. This dose produces Dot6-GFP protein levels in the *TET-DOT6* strain comparable to *msn2∆msn4∆* cells. (**E**) As shown in (**A**) but for wild-type cells paired with TET-inducible *DOT6* both grown side-by-side in 50 µg/mL doxycycline before and after NaCl treatment. (**F**) Box-plot of data from (**E**) for 379 genes. All four *TET-DOT6* samples show weaker repression compared to their paired wild-type sample (*P* < 1e-7, Wilcoxon rank-sum test). Boxplots show median (line) and 0.25 and 0.75 quartiles (box), with whiskers extending from minimum to maximum excluding outliers (circles) that are <0.25 quartile–1.5× interquartile range or >0.75 quartile–1.5× interquartile range. Exact *P* values provided in Dataset EV1. Source data are available online for this figure.

To explore this, we generated mutants in which one or the other set of paralogous transcription factors was deleted. Msn2 and Dot6 represent the primary paralogs during NaCl stress (Berry and Gasch, 2008) (Figs. EV2A–D and EV3A–F). In particular, Tod6 showed only weak nuclear translocation during NaCl stress (Fig. EV2) and had little contribution to RiBi gene repression on its own (Fig. EV3). Thus, we generated one strain expressing genomically integrated Msn2-mCherry in the absence of *DOT6/TOD6*, while another strain expressed integrated Dot6-GFP in the absence of *MSN2/4*. We compared the response of Dot6-GFP or Msn2-mCherry in each mutant to wild-type cells that expressed both Msn2-mCherry and Dot6-GFP, as well as a third constitutive fluorescence marker (Nhp6a-iRFP, see “Methods”). This enabled mixing each mutant with the wild-type and distinguishing strains based on Nhp6a-iRFP, providing a sensitive comparison of strain behaviors in the same microfluidics chamber.

**Figure 3:**
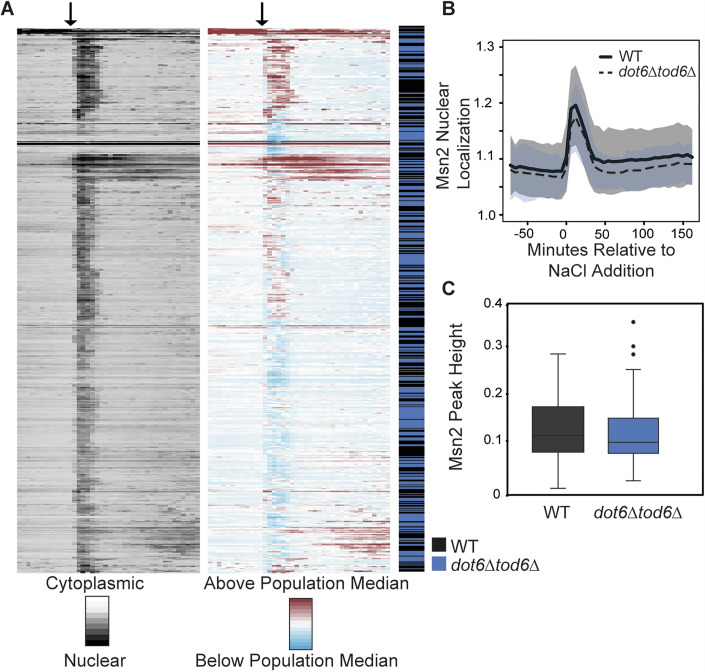
Msn2 behavior is not affected by a loss of Dot6 and Tod6. (**A**) Left: Nuclear/cytoplasmic ratio was plotted for *n* = 559 cells (wt, 272; *dot6∆tod6∆*, 287) as rows across timepoints (columns) before and after salt addition, indicated by the arrow. Right: the same data shown on the left normalized to the median of each column (population median). Cells were hierarchically clustered based on population-centered Msn2 nuclear translocation dynamics, after which cell identity was mapped onto the figure, indicating wild-type (black) or *dot6∆tod6∆* (blue) cells. (**B**) The average Msn2 nuclear/cytoplasmic ratio in wild-type (black line) and *dot6∆tod6∆* (dashed line) cells +/− one standard deviation. (**C**) Distribution of Msn2 acute stress peak heights for wild-type (black) and *dot6∆tod6∆* (blue) cells from (**A**). *P* = 0.2, Wilcoxon rank-sum test. Boxplots show median (line) and 0.25 and 0.75 quartiles (box), with whiskers extending from minimum to maximum excluding outliers (circles) that are <0.25 quartile–1.5× interquartile range or >0.75 quartile–1.5× interquartile range. Source data are available online for this figure.

Loss of *DOT6/TOD6* did not appreciably influence Msn2-mCherry nuclear dynamics (Fig. 3A–C). Mutant and wild-type cells displayed similar distributions of Msn2 localization dynamics and Msn2-mCherry localization peak heights (Fig. 3B,C). Furthermore, clustering of the individual cells based on Msn2 dynamics revealed that the two cell types cluster together and are not distinguishable by gross differences in Msn2 behavior (Fig. 3A). We conclude that the presence of Dot6 does not significantly impact the behavior of Msn2.

**Figure 4:**
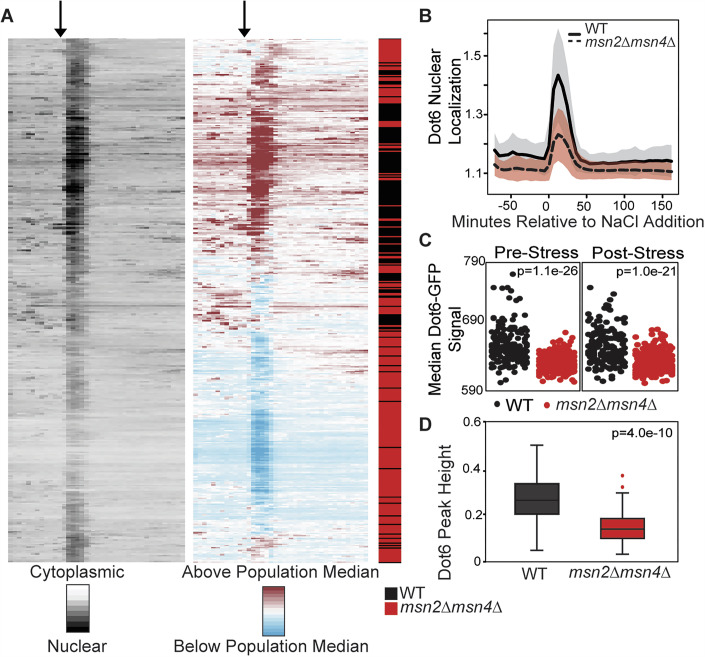
Loss of *MSN2/4* leads to decreased Dot6 abundance and nuclear localization. (**A**) Wild-type (WT) and *msn2∆msn4∆* cells from 4 replicates were clustered based on population-centered Dot6 nuclear translocation dynamics, as described in Fig. 3. (**B**) The population average of Dot6 nuclear/cytoplasmic ratio in wild-type (black line) and *msn2∆msn4∆* (dashed line) cells +/− one standard deviation. (**C**) Distribution of median Dot6-GFP signal scored before (0-72 min) or after (120–216 min) NaCl treatment (see “Methods” for details) for WT and *msn2∆msn4∆* cells; p, Wilcoxon rank-sum test. (**D**) Distribution of Dot6 acute-stress peak heights across 147 cells (WT, 43, *msn2∆msn4∆*, 104) with similar Dot6-GFP levels, see “Methods”. *P* = 2.2e-10, Wilcoxon rank-sum test. Boxplots show median (line) and 0.25 and 0.75 quartiles (box), with whiskers extending from minimum to maximum excluding outliers (circles) that are <0.25 quartile–1.5× interquartile range or >0.75 quartile–1.5× interquartile range. Source data are available online for this figure.

**Figure EV4:**
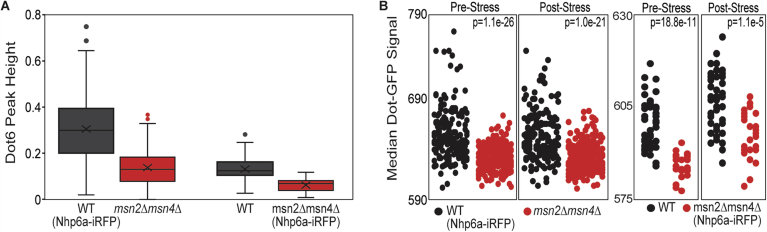
Msn2/4 effects on Dot6 activity are not an artifact of iRFP. One consideration was if iRFP expressed in one strain affected GFP signal within the same strain. To ensure that our results in Fig. 4 were not due to unanticipated effects of iRFP, we generated a new set of strains in which the *msn2∆msn4∆* cells, rather than the wild-type, carried the distinguishing iRFP signal. We found that the trends discussed in the main text were not affected by which strain carried the iRFP marker. (**A**) Distribution of Dot6 acute stress peak height across wild-type and *msn2∆msn4∆* cells when the wild-type carried expressed Nhp6a-iRFP (left, WT *n* = 173, *msn2∆msn4∆* *n* = 270) or when the *msn2∆msn4∆* strain expressed Nhp6a-iRFP (right, WT *n* = 49, *msn2∆msn4∆* *n* = 29). Despite some differences in signal for experiments done with different laser power, *msn2∆msn4∆* cells showed weaker Dot6-GFP nuclear translocation signal in both sets of experiments (*P* = 8.8e-11, left, *P* = 1.1e-5, right, Wilcoxon rank-sum test). (**B**) Distribution of median Dot6-GFP signal within the cells, scored before (0–72 min) or after (120–216 min) NaCl treatment for wild-type cells and *msn2∆msn4∆* cells as described in (**A**). p, Wilcoxon rank-sum test. Boxplots show median (line) and 0.25 and 0.75 quartiles (box), with whiskers extending from minimum to maximum excluding outliers (circles) that are <0.25 quartile–1.5× interquartile range or >0.75 quartile–1.5× interquartile range. Source data are available online for this figure.

In contrast, loss of Msn2/4 had a major impact on Dot6 behavior. Clustering cells based on Dot6-GFP translocation dynamics clearly delineated cell types: many of the *msn2∆msn4∆* cells had a weaker Dot6-GFP nuclear translocation response that was well below the median of all cells in the analysis, causing many of the *msn2∆msn4∆* cells to fall in a separate cluster (Fig. 4A). The weaker response can also be seen in the distributions of nuclear translocation dynamics, where cells lacking *MSN2/4* displayed a lower Dot6 translocation response than the wild-type (Figs. 4B and EV4A,B). We noticed that, beyond the translocation dynamics during acute stress, cells lacking *MSN2/4* showed significantly lower levels of Dot6-GFP signal overall, both before and after stress (Fig. 4C). This cannot be explained by differences in cell size (which could change signal intensity over a changing area), since *msn2∆msn4∆* cell size is indistinguishable from wild-type both before or after salt stress (*P* = 0.7 and 0.2, respectively, Wilcoxon rank-sum test). The *msn2∆msn4∆* mutant showed no difference in Tod6-GFP levels or activation, which remained weak (Fig. EV2). Thus, cells lacking *MSN2/4* have less Dot6-GFP.

**Figure EV5:**
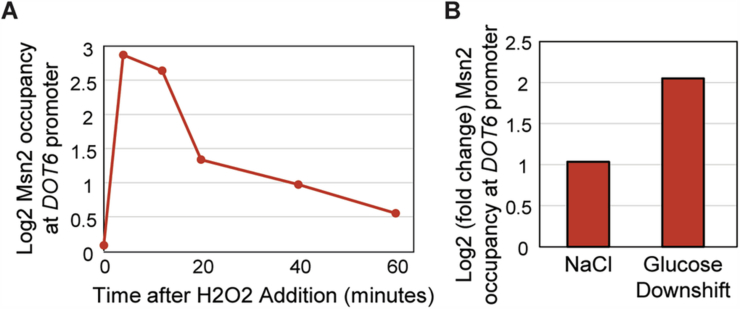
Msn2 binds *DOT6* promoter under various stresses. (**A**) Log_2_ enrichment of Msn2 occupancy relative to the whole-cell extract at the *DOT6* promoter in response to 0.4 mM H_2_O_2_ (from Huebert et al, 2012). (**B**) Log_2_ (fold change) of Msn2 occupancy at the *DOT6* promoter (ranging from 0 to −1000 bp from Ni et al and +/− 250 bp surrounding the Msn2 STRE element in the *DOT6* promoter) in response to 30 min of 0.6 M NaCl (left, from Ni et al, 2009) or 20 min after shift from glucose to glycerol (right, from Elfving et al, 2014) compared to the corresponding measurement in unstressed cells.

One question is if Msn2/4 has a direct impact on Dot6 levels/response or if the effect is indirect, perhaps simply due to the loss of iESR induction. We turned to past Msn2 chromatin-immunoprecipitation data to investigate. Remarkably, both Msn2 and Msn4 bind the Dot6 promoter after multiple stress conditions in ours and other studies (Brodsky et al, 2020; Elfving et al, 2014; Huebert et al, 2012; Kuang et al, 2017; Ni et al, 2009) (Fig. EV5A,B). The Dot6 promoter harbors one perfect-match (CCCCT) to the Msn2 binding site at -580 bp and multiple other similar C-rich sequences within ~600 bp upstream (Brodsky et al, 2020; Elfving et al, 2014), although Msn2 can localize to the promoter through protein interactions, without its binding domain (Brodsky et al, 2020; Mindel et al, 2024). These results suggest a direct conduit between Msn2 that contributes to iESR induction and regulation of Dot6 that participates in rESR repression. It is also possible that loss of *MSN2/MSN4* affects Dot6 protein levels indirectly, perhaps through degradation (Kusama et al, 2022).

We wondered if the apparent weaker nuclear translocation of Dot6-GFP in this mutant is artifactually influenced by having less GFP signal (see “Methods”). To test this, we investigated a subset of *msn2∆msn4∆* and wild-type cells who’s starting Dot6-GFP abundance was in the same range. Across 147 wild-type and *msn2∆msn4∆* cells with indistinguishable levels of Dot6-GFP (*P* = 0.5, Wilcoxon test, see “Methods”), the *msn2∆msn4∆* cells displayed significantly smaller Dot6-GFP nuclear translocation peak heights (Fig. 4D, *P* = 2.2e-10, Wilcoxon test). Thus, cells lacking *MSN2/4* have lower Dot6 abundance overall, possibly through both direct regulation of *DOT6* transcription and other indirect effects, and weaker Dot6 activation during salt stress, likely indirectly.

### Msn2/4 influences both iESR induction and rESR repression

To further explore the interplay between Msn2/4 and Dot6/Tod6, we followed dynamic changes to the transcriptome before and in ten-minute increments after salt stress in the different strains. We first defined RiBi targets under our growth conditions, identifying 489 genes whose response to salt treatment was altered in the *dot6∆tod6∆* strain compared to wild-type in at least two timepoints (FDR < 0.05), Fig. EV3A, see “Methods”). Consistent with prior results (Ho et al, 2018; Huber et al, 2011; Kunkel et al, 2019; Kusama et al, 2022; Lippman and Broach, 2009), 82% of the affected genes showed a repression defect, and 82% of those harbored matches to the known Dot6/Tod6 consensus ‘GATGAG’ within 500 bp upstream (*P* = 3.3e-58 hypergeometric test). The remaining 18% of differentially expressed genes were induced by salt, but these generally showed subtle differences (and in some cases greater induction) compared to wild-type. As expected based on the microscopy data, deletion of *TOD6* alone had little effect on expression, whereas deletion of *DOT6* produced a repression defect that was exacerbated in the absence of both regulators (Fig. EV3B,C).

**Figure 5:**
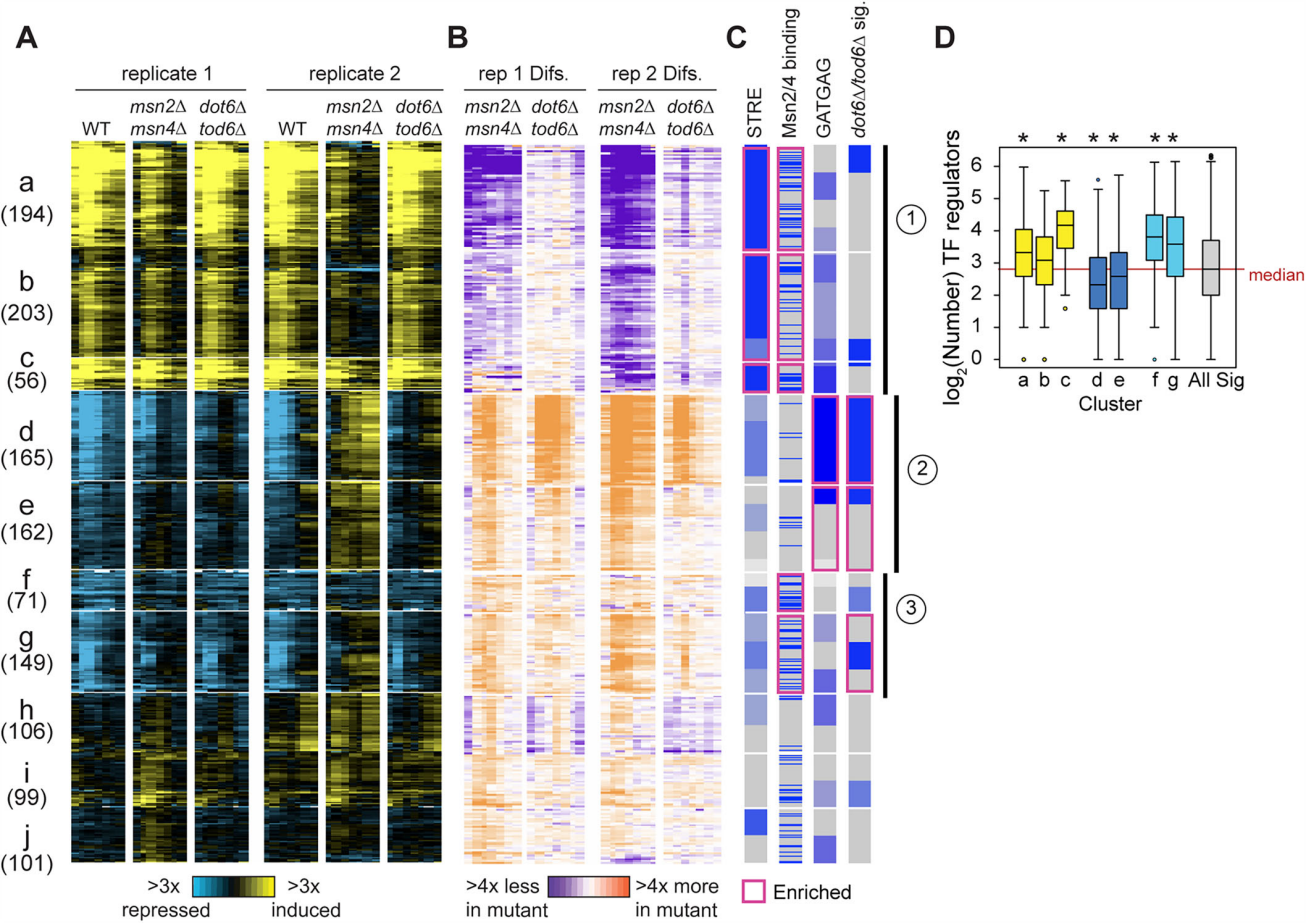
Msn2/4 influence iESR induction and rESR repression. (**A**, **B**) In total, 1306 genes (rows) differentially expressed between wild-type and *msn2∆msn4∆* cells (FDR < 0.05) in each timepoint (columns) are shown, for 10 clusters (a–j) identified by k-means clustering, where cluster size is indicated in parentheses. (**A**) Values represent log_2_(change) in expression compared to unstressed cells (blue-yellow plot) or (**B**) the log_2_(difference) in fold-change values in each mutant compared to wild-type cells, according to the keys. (**C**) Blue lines aligned with each gene from (**A**, **B**) indicate upstream STRE or GATGAG sequences, genes whose promoters are physically bound by Msn2/4, and genes whose expression is defective in the *dot6tod6∆* mutant (FDR < 0.05). Features enriched with statistical significance (FDR < 0.02, hypergeometric test) are outlined in magenta. Circled numbers represent discussion points in the text. (**D**) Distributions of log_2_ of the number of promoter-binding transcription factor regulators from Yeastract for genes in each cluster from (**A**) and 3504 genes with salt-responsive expression changes in the wild-type (‘All Sig’, FDR < 0.05) at two timepoints, see “Methods”. *FDR < 0.05, Wilcoxon rank-sum test compared to all significant genes, exact *P* values provided in Dataset EV1. The median of all significant genes is highlighted with a red line. Boxplots show median (line) and 0.25 and 0.75 quartiles (box), with whiskers extending from minimum to maximum excluding outliers (circles) that are <0.25 quartile–1.5× interquartile range or >0.75 quartile–1.5× interquartile range. Source data are available online for this figure.

In contrast to *DOT6/TOD6*, loss of *MSN2/4* produced broader expression effects. We partitioned 1306 genes significantly affected by *MSN2/4* deletion (FDR < 0.05, see “Methods”) into 10 groups by k-means clustering (Fig. 5A,B). Three of the clusters showed little expression change in wild-type cells but were weakly induced in the absence of *MSN2/4* (Fig. 5A,B, Clusters h-j), likely due to indirect effects. Three other clusters (a-c) were induced in wild-type cells but at reduced levels in the *msn2∆msn4∆* strain (Fig. 5A–C, point 1). As expected, these clusters were heavily enriched for genes with Msn2/4 binding elements within 500 bp upstream of the gene (‘STRE’, CCCCT) and for genes whose promoters are physically bound by Msn2 and/or Msn4 in response to multiple stresses including NaCl, as summarized in the Yeastract database (Elfving et al, 2014; Huebert et al, 2012; Kuang et al, 2017; Ni et al, 2009; Teixeira et al, 2023). The clusters were distinguished from one another by subtle differences in expression patterns that correlated with regulatory architecture. For example, in addition to Msn2/4 targets, Cluster a was enriched for targets of other environment-responsive factors (many of which regulate only subsets of these genes, Dataset EV2) (Gasch, 2002a; Gasch et al, 2000). Cluster b was enriched for metabolic genes involved in respiration and carbon metabolism, as well as targets of Msn2/4 and of several metabolic regulators, including Hap4, Stb5, and Oaf1 (Dataset EV2). Cluster c was very strongly induced in wild-type cells and only slightly dependent on Msn2/4 for induction; this group was also enriched for targets of many environmentally responsive regulators, especially of osmo-responsive factors Hot1 and Sko1 that can super-induce osmo-responsive genes beyond Msn2/4-regulated levels, specifically during osmotic stress (Gasch, 2002a; Gasch et al, 2000; Rep et al, 1999). Collectively, promoters of genes in clusters a and c are bound by more regulators than the average salt-responsive gene (FDR < 0.05, Wilcoxon rank-sum test, Fig. 5D). This is consistent with the known combinatorial fine-tuning of the genes in the ESR: although Msn2/4 provide a general backdrop for iESR gene induction, condition-specific regulators converge to modulate expression of subsets of genes depending on demands (Gasch, 2002a; Gasch et al, 2000).

Previous studies reported a repression defect in *msn2∆msn4∆* cells, but whether this was indirectly due to a defective stress response was not clear (Chasman et al, 2014; Elfving et al, 2014). Msn2/4 bind the *DOT6* promoter after multiple stresses, including salt stress (Elfving et al, 2014; Huebert et al, 2012; Kuang et al, 2017; Ni et al, 2009), and we showed here that Msn2/4 are required for normal Dot6 protein levels (Fig. 4A–D). Thus, we hypothesized that the *msn2∆msn4∆* strain would have a defect in *DOT6* mRNA abundance and, in turn, Dot6-dependent gene repression. Indeed, the *msn2∆msn4∆* strain had a significant defect repressing genes dependent on Dot6, which fell into Clusters d and e (Fig. 5A–C, Clusters, point 2). The *TOD6* gene (but not *DOT6*) belongs to Cluster d and harbors three upstream GATGAG sequences in its promoter, suggesting that it may be a direct target of Dot6/Tod6 repression. Clusters d and e were both heavily enriched for upstream GATGAG sequences, but no other transcription factor targets aside of general regulator Abf1 for Cluster d that is known to influence RiBi genes (Bosio et al, 2017a; Bosio et al, 2017b) (we note that known RiBi regulator Stb3 (Huber et al, 2011; Liko et al, 2007, 2010) is not included in Yeastract). In fact, clusters d and e comprised genes with a dearth of annotated transcriptional regulators compared to the total set of salt-responsive genes (Fig. 5D). Msn2/4 do not bind most of the promoters of Clusters d and e genes, and Cluster d is actually statistically significantly under-enriched for genes with upstream STRE elements (*P* = 1e-3, hypergeometric test), supporting the model that Msn2/4 dependence is indirect, perhaps in part via direct Dot6 regulation.

**Figure 6:**
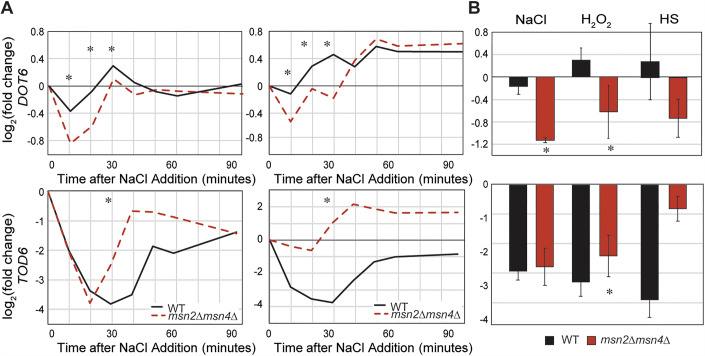
Msn2/4 are required to maintain *DOT6* mRNA levels after salt stress. (**A**) Relative log_2_(fold change) in *DOT6* (top) or *TOD6* (bottom) mRNA abundance at indicated time points compared to unstressed cells in wild-type (black) and *msn2∆msn4∆* cells (red) in replicate 1 (left) and 2 (right) RNA-seq NaCl time courses. Asterisk, significant expression difference, FDR < 0.05, *edgeR* analysis. (**B**) Average and standard deviation (*n* = 3–5) of *DOT6* (top) and *TOD6* (bottom) log_2_(fold change) in response to 30 min of 0.7 M NaCl (Chasman et al, 2014), 30 min of 0.4 mM H_2_O_2_ (Huebert et al, 2012), or 20 min after a 25–37 °C heat shock (Gasch et al, 2000). *P < 0.05, two-tailed *T* test. Exact *P* values are listed in Dataset EV1.

Msn2/4 are well-known and characterized transcriptional activators, and both move to the *DOT6* promoter after NaCl and other stresses. We found that wild-type cells maintain *DOT6* mRNA after multiple stresses, whereas the *msn2∆msn4*∆ cells expressed significantly less *DOT6* mRNA after NaCl, studied here as well as heat, peroxide stress, and glucose starvation studied previously (Elfving et al, 2014; Gasch et al, 2000; Huebert et al, 2012) (Fig. 6A,B). Given that Msn2/4 bind the *DOT6* promoter after stress, affect both *DOT6* mRNA and Dot6 protein levels, and are necessary for normal repression of Dot6 targets, we propose that the most parsimonious explanation is that Msn2/4 counteract repressive features to maintain *DOT6* expression. Because Msn2/4 can bind the *DOT6* promoter even without direct DNA binding, we were unable to ablate binding at the upstream STRE element. We did, however, generate a mutant in which genomic expression of *DOT6* was controlled by a TET-inducible promoter. Cells expressing reduced Dot6 protein levels matching those seen in the *msn2∆msn4∆* strain produced a defect in RiBi gene repression (Fig. EV3D–F). Msn2/4 did not have the same impact on *TOD6:* the gene was strongly repressed with RiBi genes in the wild-type responding to multiple stresses and showed weaker repression in the *msn2∆msn4∆* mutant, as did other RiBi genes. Furthermore, Msn2/4 are not known to bind its promoter. While evidence suggests that Msn2/4 directly regulates *DOT6*, additional indirect effects may also be at play.

In the course of analysis, we realized that loss of *MSN2/4* affected other salt-repressed genes, most notably those in Clusters f and g (Fig. 5A–C, point 3). Cluster f was enriched for genes linked to amino acid transport, methionine synthesis, and the cell periphery (FDR < 0.02, gProfiler (Kolberg et al, 2023)), whereas Cluster g comprised additional rESR genes, including several affected by Dot6/Tod6 deletion and harboring upstream GATGAG elements as well as a subset of ribosomal protein genes and others in the rESR (Fig. 5A–C, point 3). In contrast to the above repressed clusters, these groups were statistically significantly enriched for genes whose promoters are bound by Msn2/4 after multiple stresses (FDR < 0.02, (Elfving et al, 2014; Huebert et al, 2012; Kuang et al, 2017; Ni et al, 2009)). Surprisingly, however, despite increased upstream Msn2/4 binding, there was no enrichment of upstream STRE elements; in fact Cluster g was strongly under-enriched for genes with upstream STRE sequences (*P* = 3.7e-7, hypergeometric test). This was in stark contrast to induced Msn2/4 targets in clusters a–c (Fig. 5A–C, point 1) that are enriched for both Msn2/4 promoter binding and upstream binding elements. As cited above, Msn2 can bind DNA and regulate gene expression independent of its binding element or even its DNA-binding domain, through molecular interactions with its intrinsically disordered domains (Brodsky et al, 2020; Mahendrawada et al, 2025; Mindel et al, 2024). Our results suggest that repression of these genes, including many in the rESR, is affected by Msn2/4 but through a different mechanism than at iESR genes, perhaps one modulated strictly through protein interactions, which will require future dissection to elucidate.

### Modeling suggests evolutionary pressures that maintain the Msn2/4 response

Our results confirm and expand that Msn2/4 contribute to both iESR and rESR expression changes (Chasman et al, 2014; Elfving et al, 2014). The Msn2/4 response comes at a cost to growth rate during a single-stress exposure, since the *msn2∆msn4∆* mutant grows faster than wild-type in all single-stress treatments tested (Fig. 1B–D). This raises questions about the environmental pressures required to maintain Msn2/4 over evolutionary time. One pressure would be a yet-uncharacterized condition in which *msn2∆msn4∆* cells grow more slowly than wild-type. In this scenario, as long as the average wild-type growth rate weighted by exposure time is greater than that of *msn2∆msn4∆* cells, the wild-type will dominate (Abreu et al, 2020). But another possibility explaining the maintenance of the genes is through acquired resistance to a severe secondary stress, where *msn2∆msn4∆* cells have a clear defect (Fig. 2B, Berry et al, 2008). An important question is the frequency and severity of otherwise lethal secondary stress events that would be required for evolutionary maintenance of the Msn2/4 response; however, studying this in nature is challenging.

To explore this landscape, we applied a modified Lotka-Volterra competition model, frequently used to characterize competitive dynamics of mixed microbial populations growing in fixed nutrients (Bucci et al, 2016; Davis et al, 2022; Dimas Martins and Gjini, 2020; Stein et al, 2013). Although we make several simplifying assumptions (including that cells are continuously grown in single-stress conditions), this model can produce ecological bounds under which wild-type cells are more fit than *msn2∆msn4∆* cells. We adapted a published piecewise Lotka-Volterra competition model (Hsu and Zhao, 2012) to contrast the relative fitness benefit of *msn2∆msn4∆* cells growing in a single-stress condition against the relative fitness defect of *msn2∆msn4∆* cells surviving severe secondary stress. This model assumes a fixed carrying capacity, such that cells experience bouts of exponential growth and a stationary phase in the single-stress condition. We used parameters from our experimental results, including that the wild-type grows at ~0.9× the rate of the mutant in single-stress conditions (see Fig. 1D).

**Figure 7:**
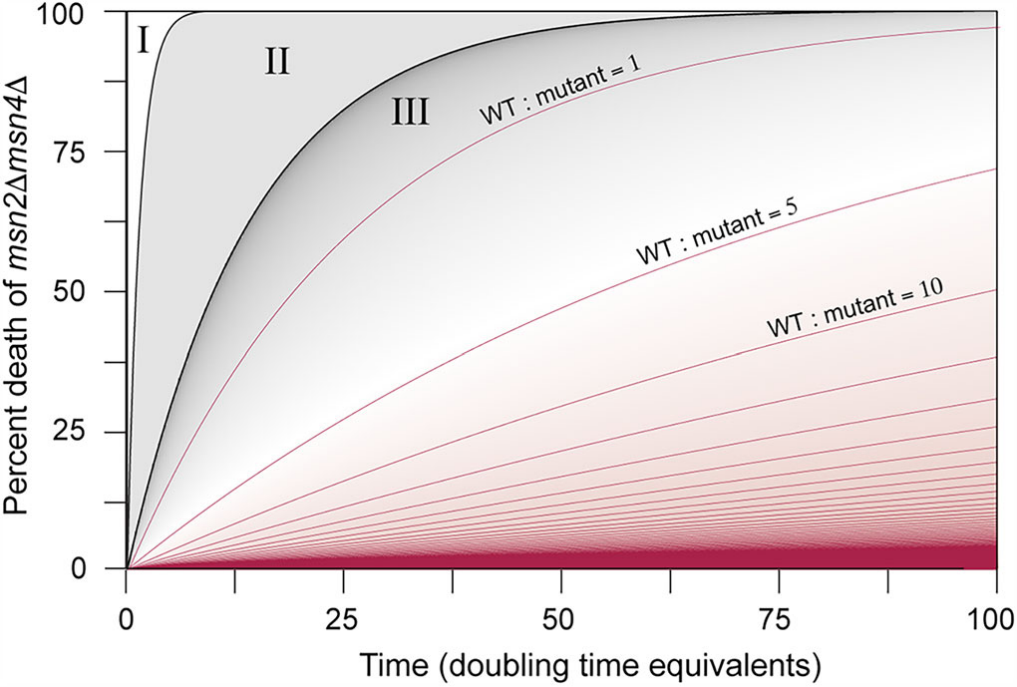
Modeling competition dynamics suggests environmental pressures that maintain the Msn2/4 response. A phase-state map based on the frequency (in doubling-time equivalents, x-axis) and severity (percent mutant killed, y-axis) of secondary stress, for conditions in which wild-type cells grow at 0.9X the *msn2∆msn4∆* growth rate, see Methods and main text for details. Competition in state III zones is dependent on the starting populations of wild-type versus mutant cells. Representative contour lines represent parameter space for different starting ratios of wild-type to mutant cells, where area above each line indicates parameters in which wild-type has the competitive advantage for that population makeup. Source data are available online for this figure.

The resulting phase map reveals the competitive dynamics of the two strains depending upon frequency (in doubling-time equivalents, *x*-axis) and severity (in percent mutant death, *y*-axis) of severe secondary stress (Fig. 7). Three states emerge, shown with defining parameters for a relative wild-type growth rate of 0.9×. At frequent and/or severe secondary stress treatments, mutant cells either die entirely (state I) or are outcompeted by wild-type (state II), regardless of the relative cell numbers in the culture. State III represents parameter zones in which competition depends on the starting proportion of wild-type and mutant cells. For example, for a starting 1:1 mixed population growing in single-stress conditions, the culture would need to experience 80% *msn2∆msn4∆* killing every 50 doubling-time equivalents for the wild-type to outcompete. However, a more realistic scenario is an excess of wild-type cells if the gene losses emerge through new mutation. At a wild-type ratio of 25:1, only 25% of *msn2∆msn4∆* cells need to die off every 100 doubling-time equivalents for wild-type cells to maintain the advantage. Periodic growth in “non-stress” conditions in which the mutant has no advantage would further reduce these requirements compared to our model. Given that *S. cerevisiae* experiences fluctuating environments in nature, such as decomposing fruit and insect-based relocation (Goddard and Greig, 2015; Jouhten et al, 2016), it is feasible that infrequent exposure to severe stresses is enough to provide the evolutionary pressure to maintain the Msn2/4 response.

## Discussion

**Figure 8:**
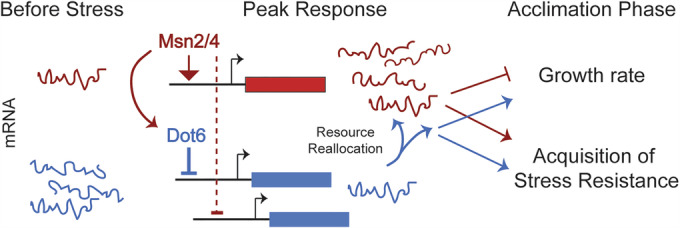
Model for Msn2-dependent resource reallocation during stress. Msn2/4 activation induces defense genes (red) and *DOT6* by direct regulation, while also influencing stress-dependent repression of ribosome and growth genes (blue), both directly through promoter binding and indirectly by influencing Dot6 activity. Tod6 may contribute to RiBi repression under other stress conditions, but did not have a significant impact in this study. Resource reallocation provided by transient rESR repression enables and accelerates the costly Msn2/4 response, promoting faster acquisition of subsequent stress tolerance, see text for details.

While it was well appreciated that stressed yeast mounts the common ESR response, understanding of the separable roles of iESR induction and rESR repression has remained incomplete. Our results here decouple these responses to quantify the importance of resource reallocation that enables the induced component of the stress response (Fig. 8). Upon acute stress, Msn2/4 transcriptionally induce their targets, most likely through direct binding of upstream STRE elements enriched in the genes’ promoters. Msn2/4 also influences rESR repression, likely through both direct effects on *DOT6* and other rESR genes through promoter binding, and indirect effects. Thus, Msn2/4 influence iESR induction and rESR repression, observed in prior studies (Chasman et al, 2014; Elfving et al, 2014). Defective rESR repression (e.g., through *DOT6/TOD6* deletion) in the context of an otherwise normal Msn2/4 response (see Fig. 1) produced a drag on post-stress growth rate, delayed production of defense proteins, and slowed acquisition of stress tolerance. Thus, we argue that the effect of Msn2/4 on rESR genes helps to coordinate resource reallocation needed for its action on induced genes.

The insights uncovered here likely pertain to stress defense systems in other organisms as well. This includes the so-called stringent response in bacteria, where the alarmone metabolite ppGpp suppresses transcription of growth-promoting genes while supporting synthesis of proteins needed for survival (Gourse et al, 2018; Zhu et al, 2019). Similar themes emerge in the mammalian integrated stress response (ISR), where a host of kinases, each responding to different signs of adversity, phosphorylate eIF2α to inhibit global translation but stimulate production of stress-responsive transcription factors that induce downstream defense targets (Costa-Mattioli and Walter, 2020; Dever et al, 2023; Harding et al, 2003; Houston et al, 2020). Results from our study in yeast can therefore reflect generalizable insights into stress responses across species.

The first is that cells alternate between objectives to balance the cost of defense versus growth. Under the conditions studied here, cells are clearly not maximizing post-stress growth rate after a single stress treatment, since they are capable of growing faster in the absence of an Msn2/4 response (Fig. 1B,C). This adds to a growing body of evidence that maximizing growth is not a universal objective in microbes, especially during adversity (Balakrishnan et al, 2021; Basan, 2018; Basan et al, 2020; Dai et al, 2018; Ho et al, 2018; Korem Kohanim et al, 2018; Schuetz et al, 2012; Zhu et al, 2024). It also shows that mere abundance of ribosome-related transcripts does not predict “instantaneous” growth rate, since cells with (albeit transiently) fewer RiBi transcripts grow faster after salt stress (Fig. 2 and Bergen et al, 2022).

Instead of maximizing growth at all costs, cells invest in preparing for impending stress at the first signs of adversity. Activation of Msn2/4 contributes to acquired stress resistance in yeast and other fungi (Berry et al, 2011; Berry and Gasch, 2008; Brown et al, 2014; Gasch, 2007; Liang et al, 2023). Similarly, activation of the bacterial stringent response supports acclimation to suboptimal carbon sources by shortening the lag phase required to produce needed proteins (Balakrishnan et al, 2021; Boutte and Crosson, 2013; Gourse et al, 2018; Zhu and Dai, 2023). Upon a shift away from optimal carbon sources, both yeast and bacteria invest in producing enzymes for alternate sugar utilization, even when the substrate sugars of those enzymes are not present (Balakrishnan et al, 2021; Simpson-Lavy and Kupiec, 2019; Turcotte et al, 2010; Vermeersch et al, 2022). Activating these responses comes with a cost to growth rate, explaining why maximal stress tolerance is not constitutive in these organisms (Balakrishnan et al, 2021; Basan et al, 2020; Zhu et al, 2024; Zhu and Dai, 2023). But it also underscores the importance of anticipatory programs in evolution and reveals a unifying theme for fast-growing microbes like *S. cerevisiae* and *E. coli*: when times are good, cells direct resources to support maximal growth, but in response to early signs of adversity, they redirect focus to invest in the future. An important corollary of this result is that growth rate is not a universal proxy for fitness in nature.

Second, our results delineate the importance of transcriptional and translational suppression for resource reallocation during stress. The isolated role of this suppression has been hard to study in other organisms, because it is often tightly coupled with the production of defense transcripts and proteins. Nonetheless, it has been suggested in *E. coli*. Cells lacking ppGpp grow fine without stress, but have a much longer lag when shifted to suboptimal conditions; conversely, ppGpp over-production, leading to stronger repression of growth-promoting genes, slows growth in the absence of stress but accelerates stress acclimation and promotes stress tolerance (Zhu et al, 2019, 2024; Zhu and Dai, 2023). Furthermore, over-production of unnecessary proteins slows cell growth rate and stress acclimation, supporting the notion that a tax on protein-synthesis capacity is suboptimal (Balakrishnan et al, 2021; Basan et al, 2020). The intimate coupling of stress-induced and -repressed responses across organisms mounting common stress responses underscores that coregulated resource reallocation is a unifying principle.

A remaining question had been how this resource reallocation is regulated in yeast. Our results imply that resource reallocation is built into the ESR program (Fig. 8). Msn2/4 maintain Dot6 protein levels, likely through direct binding of the *DOT6* promoter and potentially other indirect effects (Figs. 4, 6, and EV5). Our evidence suggests they also contribute to repression of other rESR promoters during stress in a manner that may be distinct from induced proteins, given the enrichment of Msn2/4 promoter binding but dearth of upstream binding elements for these gene groups (Fig. 5). Thus, Msn2/4 activity helps to orchestrate the resource reallocation needed for its own response. A remaining mystery is how yeast sense their internal system to set the balance between growth-promoting and stress-defending programs. *S. cerevisiae* does not utilize ppGpp, which in *E. coli* directly senses translational flux at individual ribosomes (Wu et al, 2022). In yeast, PKA and TOR may play a role, since they respond to quality nutrients to promote growth at the expense of defense (González and Hall, 2017; Kocik and Gasch, 2022). Even in an organism as well-studied as *S. cerevisiae*, these mysteries await further investigation.

## Methods

**Table:** Reagents and tools table

Reagent/resource	Reference or source	Identifier or catalog number
**Experimental models**		
*S. cerevisiae - MATa, his3Δ1, leu2Δ0, ura3Δ0, met15Δ0*	Open Biosystems	AGY4
*S. cerevisiae - MATα, his3Δ1, leu2Δ0, ura3Δ0, lys2Δ0*	Open Biosystems	AGY5
*S. cerevisiae - MATa, his3Δ1, leu2Δ0, ura3Δ0, met15Δ0, ctt1::KAN*	Open Biosystems	AGY345
*S. cerevisiae - MATa, his3Δ1, leu2Δ0, ura3Δ0, met15Δ0, dot6::KAN*	Open Biosystems	AGY523
*S. cerevisiae - MATa, his3Δ1, leu2Δ0, ura3Δ0, met15Δ0, tod6::KAN*	Open Biosystems	AGY524
*S. cerevisiae - MATa, his3Δ1, leu2Δ0, ura3Δ0, met15Δ0, dot6::KAN, tod6::HYG*	Lee et al, 2011	AGY594
*S. cerevisiae - MATa, his3Δ1, leu2Δ0, ura3Δ0, met15Δ0 DOT6-GFP(S65T)-His3MX, MSN2-mCherry-HYGMX*	Bergen et al, 2022	AGY1328
*S. cerevisiae - MATa, his3Δ1, leu2Δ0, ura3Δ0, met15Δ0, TOD6-GFP(S65T)-His3MX*	Open Biosystems	AGY1441
*S. cerevisiae - MATa, his3Δ1, leu2Δ0, ura3Δ0, met15Δ0, msn2::KAN, msn4::HYG*	This study	AGY2046
*S. cerevisiae - MATα, his3Δ1, leu2Δ0, ura3Δ0, met15Δ0 DOT6-GFP(S65T)-His3MX, MSN2-mCherry-HYGMX*	This study	AGY2228
*S. cerevisiae - MATα, his3Δ1, leu2Δ0, ura3Δ0, lys2Δ0, msn2::KAN, msn4::HYG*	This study	AGY2229
*S. cerevisiae - MATa, his3Δ1, leu2Δ0, ura3Δ0, met15Δ0 DOT6-GFP(S65T)-His3MX, MSN2-mCherry-HYGMX, NHP6A-iRFP*	This study	AGY2233
*S. cerevisiae - MATa, his3Δ1, leu2Δ0, ura3Δ0, met15Δ0, dot6::KAN tod6::HYG, MSN2-mCherry-HYGMX*	This study	AGY2234
*S. cerevisiae - MATα, his3Δ1, leu2Δ0, ura3Δ0, met15Δ0, MSN2-mCherry-HYGMX*	This study	AGY2237
*S. cerevisiae - MATa, his3Δ1, leu2Δ0, ura3Δ0, met15Δ0, TOD6-GFP(S65T)-His3MX, NHP6A-iRFP*	This study	AGY2242
*S. cerevisiae - MATa, his3Δ1, leu2Δ0, ura3Δ0, met15Δ0, chrV: 333177-333677::URA3*	This study	AGY2244
*S. cerevisiae - MATa, his3Δ1, leu2Δ0, ura3Δ0, met15Δ0, chrV: 333177-333677::URA3, DOT6-GFP(S65T)-His3MX*	This study	AGY2245
*S. cerevisiae - MATa, his3Δ1, leu2Δ0, ura3Δ0, met15Δ0 TOD6-GFP(S65T)-His3MX, MSN2-mCherry-HYGMX*	This study	AGY2247
*S. cerevisiae - MATa, his3Δ1, leu2Δ0, ura3Δ0, met15Δ0, msn2::KAN msn4::HYG, DOT6-GFP(S65T)-His3MX*	This study	AGY2254
*S. cerevisiae - MATa, his3Δ1, leu2Δ0, ura3Δ0, met15Δ0, msn2::KAN msn4::HYG, DOT6-GFP(S65T)-His3MX, NHP6A-iRFP*	This study	AGY2255
*S. cerevisiae - MATa, his3Δ1, leu2Δ0, ura3Δ0, met15Δ0, MYO2pr-rtTA-TETO7promoter-DOT6*	This study	AGY2256
*S. cerevisiae - MATa, his3Δ1, leu2Δ0, ura3Δ0, met15Δ0, MYO2pr-rtTA-TETO7promoter-DOT6 -GFP(S65T)-His3MX*	This study	AGY2257
*S. cerevisiae - MATa, his3Δ1, leu2Δ0, ura3Δ0, met15Δ0, msn2::KAN msn4::HYG, TOD6-GFP(S65T)-His3MX*	This study	AGY2260
*S. cerevisiae - MATa, his3Δ1, leu2Δ0, ura3Δ0, met15Δ0, msn2::KAN, msn4::HYG dot6::KAN tod6::HYG*	This study	AGY2264
**Oligonucleotides and other sequence-based reagents**		
PCR Primer (msn4::HYG forward)	This study (IDT)	5’-TATCAGTTCGGCTTTTTTTTCTTTTCTTCTTATTAAAAACGTTCGAGTTTATCATTATCAATACTGCC-3’
PCR Primer (msn4::HYG reverse)	This study (IDT)	5’-GCTTGTCTTGCTTTTATTTGCTTTTGACCTTATTTTTTTCCCGGTAGAGGTGTGGTCAATAAG-3’
PCR Primer (URA3-DOT6 forward)	This study (IDT)	5’-CCACCACCATCGCTACCAACAGCAGGATATCCGGATGCAGTTTGACGCTTTTCTGGGTAGAAGATCGGTCTG-3'
PCR Primer (URA3-DOT6 reverse)	This study (IDT)	5’-ATGCTGCTCAAATGAATGGAAGCTGAGTTCAAACTGGTTGAAATGGACATCAGTTCAATACAACAGATCACGTG-3'
PCR Primer (TET-DOT6 forward)	This study (IDT)	5’-CCACCACCATCGCTACCAACAGCAGGATATCCGGATGCAGTTTGACGCTTAACGCCGTTTCTCGATGCTTATCTG-3'
PCR Primer (TET-DOT6 reverse)	This study (IDT)	5’-ATGCTGCTCAAATGAATGGAAGCTGAGTTCAAACTGGTTGAAATGGACATGTGCTCAGTATCTCTATCACTGATA-3'
**Chemicals, enzymes, and other reagents**		
Yeast nitrogen base without ammonium sulfate, folic acid, or riboflavin	Sigma	Y1251-100G
Ammonium sulfate	Sigma	A2939-100G
Yeast synthetic drop-out medium without histidine	Sigma	Y1751
Histidine	Fisher	BP382-100
Dextrose	Fisher	D16500
Sodium chloride	Fisher	AC327300010
Hydrochloric acid	Fisher	A144SI-212
Sodium hydroxide	Fisher	S3818100
Absolute ethanol	Fisher	BP28184
Hydrogen peroxide (30% in water)	Fisher	BP2633500
Doxycycline hyclate	Sigma	D9891-5G
Zymolyase	Zymo Research	E1004 or E1005
Concanavalin A	MP Biomedical	150710
Phenol ≥99.0% saturated with buffer pH 4.5	VWR	0981-400 ML
Chloroform	Fisher	C298-500
RNase-free DNase set	Qiagen	79254
**Software**		
NIS-Elements	Nikon	n/a
MATLAB	The MathWorks, Inc.	Version R2022b
R	R Foundation, USA	Version 4.3.1
Gene Cluster 3.0	Eisen et al, 1998	n/a
Java TreeView	Saldanha, 2004	Version 1.2.0
Trimmomatic	Bolger et al, 2014	Version 0.39
Bowtie 2	Langmead and Salzberg, 2012	Version 2.4.4
HTSeq	Anders et al, 2015	Version 0.6.0
edgeR	Robinson and Oshlack, 2010	Version 4.3.2
Yeastract	Teixeria et al, 2023	n/a
RSAT	Santana-Garcia et al, 2022	n/a
**Other**		
Spectronic 20D+ spectrophotometer	2007 Thermo Fisher Scientific Inc	n/a
FCS2 chamber	Bioptechs Inc	n/a
Nikon Eclipse Ti inverted microscope with perfect focus system	Nikon	n/a
ET-EGFP single band filter cube	Chroma Technology Corp	#49002
ET/mCH/TR single band filter cube	Chroma Technology Corp	#96365
Cy 5.5 single band filter cube	Chroma Technology Corp	#49022
RNeasy MinElute Cleanup Kit	Qiagen	#74204
TruSeq Stranded mRNA Kit	Illumina	20020595
AMPure XP Beads	Fisher Scientific	NC9933872
NovaSeq 6000	Illumina	n/a

### Strains and growth conditions

*Saccharomyces cerevisiae* strains of the BY4741 background used in this study are listed in Table 1. All strains were grown in Low Fluorescent Media (LFM) as previously described in Bergen et al, 2022 (0.17% yeast nitrogen base without ammonium sulfate, folic acid, or riboflavin; 0.5% ammonium sulfate; 0.2% complete amino acid supplement, and 2% glucose). Strain AGY2046 (*msn2∆msn4∆*) was generated by replacing *MSN4* in the BY4741 *msn2::KANMX* strain (Open Biosystems) with the hygromycin-MX cassette via homologous recombination and validated using diagnostic PCRs. Strain AGY2244 and AGY2245 were generated by replacing 500 bp upstream of *DOT6* start codon in the BY4741 wild-type strain (AGY4) or AGY1319 expressing DOT6-GFP with the *K. lactis URA3* gene via homologous recombination to knock out the endogenous *DOT6* promoter region, then replacing *K. lactis URA3* upstream of the *DOT6* (or DOT6-GFP) start codon with a construct containing the Tet promoter along with the rtTA inducer (MYO2promoter-rtTA-TETO7promoter – gift from Michael Springer Lab) via homologous recombination, selecting for loss of uracil prototrophy. The strain was validated using diagnostic PCR and microscopy to verify doxycycline-responsive Dot6-GFP induction. The remaining strains were generated through genetic crosses as listed below, dissection of haploid spores, and selection of spores with appropriate markers. Gene deletions were verified by diagnostic PCR and fluorescent microscopy when appropriate. Crosses were used to generate AGY2228 (AGY1328 x AGY5), AGY2229 (AGY2046 x AGY5), AGY2233 (AGY1328 x AGY2232), AGY2234 (AGY594 x AGY2228), AGY2237 (AGY1328 x AGY5), AGY2242 (AGY1441 x AGY2232), AGY2254 (AGY1328 x AGY2229), AGY2247 (AGY1441 x AGY2237), AGY2255 (AGY2234 x AGY2232), AGY2260 (AGY1441 x AGY2229), and AGY2264 (AGY594 x AGY2229).

**Table 1:** Strains used in this study.

Strain Name	Description	Source
AGY4	*MATa, his3Δ1, leu2Δ0, ura3Δ0, met15Δ0*	Open Biosystems
AGY5	*MATα, his3Δ1, leu2Δ0, ura3Δ0, lys2Δ0*	Open Biosystems
AGY345	*MATa, his3Δ1, leu2Δ0, ura3Δ0, met15Δ0, ctt1::KAN*	Open Biosystems
AGY523	*MATa, his3Δ1, leu2Δ0, ura3Δ0, met15Δ0, dot6::KAN*	Open Biosystems
AGY524	*MATa, his3Δ1, leu2Δ0, ura3Δ0, met15Δ0, tod6::KAN*	Open Biosystems
AGY594	*MATa, his3Δ1, leu2Δ0, ura3Δ0, met15Δ0, dot6::KAN, tod6::HYG*	Lee et al, 2011
AGY1328	*MATa, his3Δ1, leu2Δ0, ura3Δ0, met15Δ0 DOT6-GFP(S65T)-His3MX, MSN2-mCherry-HYGMX*	Bergen et al, 2022
AGY1441	*MATa, his3Δ1, leu2Δ0, ura3Δ0, met15Δ0, TOD6-GFP(S65T)-His3MX*	Open Biosystems
AGY2046	*MATa, his3Δ1, leu2Δ0, ura3Δ0, met15Δ0, msn2::KAN, msn4::HYG*	This study
AGY2228	*MATα, his3Δ1, leu2Δ0, ura3Δ0, met15Δ0 DOT6-GFP(S65T)-His3MX, MSN2-mCherry-HYGMX*	This study
AGY2229	*MATα, his3Δ1, leu2Δ0, ura3Δ0, lys2Δ0, msn2::KAN, msn4::HYG*	This study
AGY2233	*MATa, his3Δ1, leu2Δ0, ura3Δ0, met15Δ0 DOT6-GFP(S65T)-His3MX, MSN2-mCherry-HYGMX, NHP6A-iRFP*	This study
AGY2234	*MATa, his3Δ1, leu2Δ0, ura3Δ0, met15Δ0, dot6::KAN tod6::HYG, MSN2-mCherry-HYGMX*	This study
AGY2237	*MATα, his3Δ1, leu2Δ0, ura3Δ0, met15Δ0, MSN2-mCherry-HYGMX*	This study
AGY2242	*MATa, his3Δ1, leu2Δ0, ura3Δ0, met15Δ0, TOD6-GFP(S65T)-His3MX, NHP6A-iRFP*	This study
AGY2244	*MATa, his3Δ1, leu2Δ0, ura3Δ0, met15Δ0, chrV:* 333177-333677*::URA3*	This study
AGY2245	*MATa, his3Δ1, leu2Δ0, ura3Δ0, met15Δ0, chrV: 333177-333677::URA3, DOT6-GFP(S65T)-His3MX*	This study
AGY2247	*MATa, his3Δ1, leu2Δ0, ura3Δ0, met15Δ0 TOD6-GFP(S65T)-His3MX, MSN2-mCherry-HYGMX*	This study
AGY2254	*MATa, his3Δ1, leu2Δ0, ura3Δ0, met15Δ0, msn2::KAN msn4::HYG, DOT6-GFP(S65T)-His3MX*	This study
AGY2255	*MATa, his3Δ1, leu2Δ0, ura3Δ0, met15Δ0, msn2::KAN msn4::HYG, DOT6-GFP(S65T)-His3MX, NHP6A-iRFP*	This study
AGY2256	*MATa, his3Δ1, leu2Δ0, ura3Δ0, met15Δ0, MYO2pr-rtTA-TETO7promoter-DOT6*	This study
AGY2257	*MATa, his3Δ1, leu2Δ0, ura3Δ0, met15Δ0, MYO2pr-rtTA-TETO7promoter-DOT6 -GFP(S65T)-His3MX*	This study
AGY2260	*MATa, his3Δ1, leu2Δ0, ura3Δ0, met15Δ0, msn2::KAN msn4::HYG, TOD6-GFP(S65T)-His3MX*	This study
AGY2264	*MATa, his3Δ1, leu2Δ0, ura3Δ0, met15Δ0, msn2::KAN, msn4::HYG dot6::KAN tod6::HYG*	This study

### Liquid growth curves

Liquid cultures for growth rate assessment were inoculated in test tubes from an overnight culture grown ~12 h in LFM and grown for at least 4.5 h to a starting optical density at 600 nm (OD_600_) of ~0.1 before measurements were taken every 15 min. For salt stress, NaCl was added to 0.7 M NaCl. For heat stress, cells were collected by centrifugation and then shifted to 40 °C media. For alkaline stress, cells were collected by centrifugation and transferred to LFM adjusted to pH 7.5. For acid stress, cells were collected by centrifugation and transferred to LFM adjusted to pH 2.75. For ethanol stress, absolute ethanol was added to 3% and 4%. Growth rates were calculated by fitting exponential curves to data from time points spanning 75 min to 225 min after NaCl was added or exponential time points for other stresses. Mutants were grown side-by-side with wild-type cultures, with paired replicates done on separate days, allowing paired statistical analysis.

### Acquired stress resistance experiments

See Fig. 2A for a schematic of this protocol. Cultures were grown in LFM in flasks at 30 °C in a shaking incubator for at least 15 h to a starting OD_600_ ~0.3–0.4. An aliquot of unstressed cells (0 min) was removed, and then NaCl was added to a final concentration of 0.7 M. At various timepoints following the addition of NaCl (10, 20, 30, 40, 50, 60, 90, and 120 min), an aliquot of culture was retrieved, cells collected by brief centrifugation, and resuspended in fresh LFM without NaCl to an OD_600_ of 0.6. Cells were subsequently threefold diluted into 96-well plates containing LFM or LFM plus one of 11 doses of H_2_O_2_ (spanning from 0 to 20 mM final concentration of H_2_O_2_). Cells were incubated for 2 h at 30 °C in a shaking incubator, then a 200-fold dilution of each culture was spotted on YPD agar plates (1% yeast extract, 2% peptone, 2% glucose, and 2% agar). Plates were grown ~48 h at 30 °C, then viability at each dose of H_2_O_2_ “secondary” stress was scored visually on a four-point scale: 100% (3), 50–100% (2), 10–50% (1), and 0% (0) survival compared to the wild-type cells treated with NaCl but no H_2_O_2_. A single H_2_O_2_ survival score was calculated for each time point as the sum of scores across the 11 different doses of H_2_O_2_. Each mutant was compared to wild-type culture grown side-by-side on each day, with 3 biological replicates for most strains except the *dot6∆tod6∆*, which was done with 6 replicates for added statistical power.

### Microscopy and image analysis

Time-lapse microscopy was performed using an FCS2 chamber (Bioptechs Inc., Butler, Pennsylvania). Data collection and analyses were conducted as previously described in Bergen et al, 2022 (Bergen et al, 2022), with the following changes. Each mutant was grown to mid-log phase in LFM media in a flask and then mixed within the microfluidic chamber at a 50:50 ratio with the iRFP-tagged wild-type strain. Media flow was switched from LFM to LFM + 0.7 M NaCl after T12, as previously described. The GFP and mCherry signal was recorded at each time point before and after NaCl treatment as previously described. To distinguish wild-type from mutant cells in mixed cultures, Principal component analysis of cells was performed based on the iRFP signal across timepoints T1-T40, using R Statistical Software (R version 4.3.1). This analysis led to a clear dichotomy of cell types that also correlated with the presence of both GFP and mCherry signal, where mutant cells showed no iRFP and loss of either GFP or mCherry signal according to the strain.

Dot6-GFP and Msn2-mCherry phenotypes were also determined as previously described, including fraction of nuclear Dot6-GFP and Msn2-mCherry signal (“nuclear/cytoplasmic ratio”, defined as the average signal of the top 5% of pixels divided by the median of all pixels), acute stress peak height, and area under the curve of fraction of nuclear signal across pre-stress or post-stress timepoints (Bergen et al, 2022). Tod6-GFP phenotypes were also calculated in the same way. Acute stress peak height as shown in Figs. 3–4 and  EV2 was calculated as the maximum nuclear localization score just after NaCl addition (T13–T20) minus the minimum fraction of nuclear signal just before salt was added (T11–T13). Dot6 and Tod6 abundance was measured based on the median Dot6-GFP or Tod6-GFP signal in each cell; the average signal before NaCl treatment (T1–T12) or after (T20–T36) is shown in Figs. 4C and  EV2B, respectively. Cells shown in Fig. 4D were defined as those with a similar levels of Dot6-GFP signal (values between 635 to 650 signal intensity), such that the mutant and wild-type signal were not different (Wilcoxin rank-sum test *P* > 0.05). Cells shown in Fig. EV2C were defined as those with similar levels of Tod6-GFP signal (values between 605 and 615 signal intensity), such that mutant and wild-type signals were not different (Wilcoxon rank-sum test *P* > 0.05).

Cell clustering in Figs. 3–4 was performed based on the population median (i.e., each column) of GFP or mCherry nuclear signal using Gene Cluster 3.0 (Eisen et al, 1998) and visualized using Java TreeView version 1.2.0 (Saldanha, 2004). The fraction of nuclear signal shown on the left was added after clustering for display. Data are available in Dataset EV3.

### RNA sequencing

RNA-seq was performed using total RNA isolated from log-phase cultures before and after NaCl treatment. Cultures were grown in LFM in flasks at 30 °C in a shaking incubator for at least 16 h to a starting OD_600_ of mid-log phase. For TET-inducible experiments, all cells being compared were grown in LFM + 50 µg/mL Doxycycline (Sigma-Aldrich) for 16 h. 5 mL of culture was harvested via centrifugation at 3000 RPM for 3 min, flash frozen in liquid nitrogen, and stored at −80 °C. Total RNA was extracted using hot phenol lysis (Gasch, 2002b) and purified using the RNeasy MinElute Cleanup Kit (QIAGEN, Hilden, Germany) and DNase digestion. RNA-seq libraries were prepared using a TruSeq Stranded Total RNA kit (Illumina), and PCR purified using AMPure XP beads (Beckman Coulter, Indianapolis, IN). Paired-end sequencing was performed on an Illumina NovaSeq 6000 sequencer (Illumina).

RNA-seq reads were processed using Trimmomatic version 0.39 (Bolger et al, 2014) and mapped to the S288c genome using Bowtie 2 version 2.4.4 (Langmead and Salzberg, 2012). Read counts for each gene were calculated using HTSeq version 0.6.0 (Anders et al, 2015). Raw data can be found in the NIH GEO database Accession GSE283327. Differentially expressed genes were identified at each timepoint using a glm model in edgeR version 4.3.2 using TMM normalization (Robinson and Oshlack, 2010) with significance at <0.05 Benjamini and Hochberg false discovery rate (FDR) (Benjamini and Hochberg, 1995). Genes significant in at least two time points were considered for analysis. Cells with an induction (or repression) defect were defined if the gene was induced (or repressed) in a majority of time points in the wild-type cells and showed a smaller log_2_(fold change) in the corresponding mutant. Data are available in Dataset EV4.

In total, 1306 genes with significant differences in salt-induced expression in the *msn2∆msn4∆* strain were partitioned into *k* = 10 clusters by k-means clustering (where k was defined manually) based on the log_2_(fold change) in expression in wild-type, *msn2∆msn4∆*, and *dot6∆tod6∆* time courses, along with the log_2_ differences at each timepoint in each mutant versus wild-type (as shown in Fig. 5A,B). Targets of 184 transcription factors with evidence of direct DNA binding were received courtesy of Yeastract (Teixeira et al, 2023) and used to score enrichment in each cluster compared to all yeast genes, using the hypergeometric test and Benjamini-Hochberg correction. Upstream STRE (CCCCT) or GATGAG sequences were scored within 500 bp upstream of genes in each cluster, searching both strands and preventing overlapping hits, using RSAT (Santana-Garcia et al, 2022).

### Modeling

To explore the necessary frequency and severity of secondary stress treatments required to provide wildtype cells with an advantage, we constructed the following piecewise Lotka-Volterra model, assuming that the yeast grow exponentially with linear competition until saturation, in a culture that can support a fixed number of cells (i.e., which has a fixed carrying capacity). We assumed both the carrying capacities and mutual competition of the two strains to be equivalent, as Msn2/4 are not known to have an effect on these traits. We further simplified by modeling only a single-stress growth condition in which the wild-type grows more slowly and where wild-type death during secondary stress is discounted, since it is much less than that of the mutant. The first assumption makes our model more stringent than is likely in nature. These assumptions yield the following model

$$\frac{d{N}_{{WT}}}{{dt}}=\left\{\begin{array}{cc}{r}_{{WT}}{N}_{{WT}}(1-\frac{{N}_{{WT}}+{N}_{{Null}}}{K}) & m\omega +(1-\phi )\omega \le t\le (m+1)\omega \\ 0\hfill & m\omega \le t\le m\omega +(1-\phi )\omega \hfill\end{array}\right.$$

$$\frac{d{N}_{{Null}}}{{dt}}=\left\{\begin{array}{cc}{r}_{{Null}}{N}_{{Null}}(1-\frac{{N}_{{WT}}+{N}_{{Null}}}{K}) & m\omega +(1-\phi )\omega \le t\le (m+1)\omega \\ -D{N}_{{Null}}\hfill & m\omega \le t\le m\omega +(1-\phi )\omega \hfill\end{array}\right.$$

where ${N}_{{WT}}$ is the number of wildtype cells and ${N}_{{Null}}$ is the number of *msn2∆msn4∆* cells, with ${r}_{{WT}}$ and ${r}_{{Null}}$ as their respective growth rates. K is the carrying capacity, $D$ is the death rate of *msn2∆msn4∆* cells in secondary stress conditions, ${{\rm{\omega }}}$ is the period of secondary stress cycles with ${{\rm{\omega }}}\left(1-{{\rm{\phi }}}\right)$ the duration of the resulting severe stress.

This in turn enables us to use a modified version of the results in Hsu and Zhao, 2012, deriving the following possible states depending on the frequency and severity of secondary stresses:

State I: The *msn2∆msn4∆* death per secondary-stress exposure is greater than recovered by their growth between secondary stress events, such that all *msn2∆msn4∆* cells die regardless of competition.

State II: Sufficient *msn2∆msn4∆* growth to compensate for death occurs between secondary stress events, however, they are outcompeted by wild-type cells regardless of relative initial proportions of the two strains in the population.

State III: Either the *msn2∆msn4∆* cells or the wildtype cells can outcompete the other, depending on relative initial proportions of the two strains.

We can then reparametrize the above equations in terms of the relative fitness $\alpha =\frac{{r}_{{WT}}}{{r}_{{Null}}}$, time between secondary stresses normalized by the uninhibited doubling time $\beta =\frac{{{\rm{\phi }}}}{{ln}\left(2\right)}$, and the mutant mortality following each secondary stress exposure $\frac{{Deat}{h}_{{Null}}}{{N}_{{Null}}}=1-{e}^{-D\left(1-{{\rm{\phi }}}\right)}$. For ${r}_{{Null}}=1$, the states are defined by the following formulae:

State I: *msn2∆msn4∆* cells are nonviable if

$$\frac{{Deat}{h}_{{Null}}}{{N}_{{Null}}} > 1-{e}^{-{{\rm{\beta }}}{ln}\left(2\right)}$$

State II: Wild-type cells always outcompete *msn2∆msn4∆* cells if

$$\frac{{Deat}{h}_{{Null}}}{{N}_{{Null}}} > 1-{e}^{\left(\alpha -1\right)\beta {\mathrm{ln}}\left(2\right)}$$

Otherwise, wild-type cells outcompete *msn2∆msn4∆* cells so long as

$$\frac{{N}_{{WT}}}{{N}_{{Null}}} > \frac{\left(1-\frac{1}{{{\rm{\alpha }}}}\right){{\rm{\beta }}}\,{ln}\left(2\right)}{{ln}\left(1-\frac{{Deat}{h}_{{Null}}}{{N}_{{Null}}}\right)}-\frac{1}{{{\rm{\alpha }}}}$$

## Supplementary information

Peer Review File

Dataset EV1

Dataset EV2

Dataset EV3

Dataset EV4

Source data Fig. 1

Source data Fig. 2

Source data Fig. 3

Source data Fig. 4

Source data Fig. 5

Source data Fig. 7

Figure EV1 Source Data

Figure EV2 Source Data

Figure EV3 Source Data

Figure EV4 Source Data

Expanded View Figures

## Data Availability

The RNA-seq data from this publication have been deposited to the NIH GEO database https://www.ncbi.nlm.nih.gov/geo/] and assigned the identifier #GSE283327. The source data of this paper are collected in the following database record: biostudies:S-SCDT-10_1038-S44318-026-00727-x.

## References

[CR1] Abreu CI, Andersen Woltz VL, Friedman J, Gore J (2020) Microbial communities display alternative stable states in a fluctuating environment. PLoS Comput Biol 16:e1007934 32453781 10.1371/journal.pcbi.1007934 PMC7274482

[CR2] Anders S, Pyl PT, Huber W (2015) HTSeq—a Python framework to work with high-throughput sequencing data. Bioinformatics 31:166–169 25260700 10.1093/bioinformatics/btu638 PMC4287950

[CR3] Badis G, Chan ET, van Bakel H, Pena-Castillo L, Tillo D, Tsui K, Carlson CD, Gossett AJ, Hasinoff MJ, Warren CL et al (2008) A library of yeast transcription factor motifs reveals a widespread function for Rsc3 in targeting nucleosome exclusion at promoters. Mol Cell 32:878–887 19111667 10.1016/j.molcel.2008.11.020 PMC2743730

[CR4] Bagamery LE, Justman QA, Garner EC, Murray AW (2020) A putative bet-hedging strategy buffers budding yeast against environmental instability. Curr Biol 30:4563–4578 32976801 10.1016/j.cub.2020.08.092 PMC7726042

[CR5] Balaban NQ, Merrin J, Chait R, Kowalik L, Leibler S (2004) Bacterial persistence as a phenotypic switch. Science 305:1622–1625 15308767 10.1126/science.1099390

[CR6] Balakrishnan R, de Silva RT, Hwa T, Cremer J (2021) Suboptimal resource allocation in changing environments constrains response and growth in bacteria. Mol Syst Biol 17:e10597 34928547 10.15252/msb.202110597 PMC8687047

[CR7] Barber F, Min J, Murray AW, Amir A (2021) Modeling the impact of single-cell stochasticity and size control on the population growth rate in asymmetrically dividing cells. PLoS Comput Biol 17:1–17 10.1371/journal.pcbi.1009080 PMC8248971 34153030

[CR8] Basan M (2018) Resource allocation and metabolism: the search for governing principles. Curr Opin Microbiol 45:77–83 29544124 10.1016/j.mib.2018.02.008

[CR9] Basan M, Honda T, Christodoulou D, Hörl M, Chang Y-F, Leoncini E, Mukherjee A, Okano H, Taylor BR, Silverman JM et al (2020) A universal trade-off between growth and lag in fluctuating environments. Nature 584:470–474 32669712 10.1038/s41586-020-2505-4 PMC7442741

[CR10] Basu S, Dong Y, Kumar R, Jeter C, Tang DG (2022) Slow-cycling (dormant) cancer cells in therapy resistance, cancer relapse and metastasis. Semin Cancer Biol 78:90–103 33979674 10.1016/j.semcancer.2021.04.021 PMC8576068

[CR11] Beck T, Hall MN (1999) The TOR signalling pathway controls nuclear localization of nutrient-regulated transcription factors. Nature 402:689–692 10604478 10.1038/45287

[CR12] Benjamini Y, Hochberg Y (1995) Controlling the false discovery rate: a practical and powerful approach to multiple testing. J R Stat Soc Ser B Methodol 57:289–300

[CR13] Bergen AC, Kocik RA, Hose J, McClean MN, Gasch AP (2022) Modeling single-cell phenotypes links yeast stress acclimation to transcriptional repression and pre-stress cellular states. eLife 11:e82017 36350693 10.7554/eLife.82017 PMC9678356

[CR14] Bergenholm D, Liu G, Holland P, Nielsen J (2018) Reconstruction of a global transcriptional regulatory network for control of lipid metabolism in yeast by using chromatin immunoprecipitation with lambda exonuclease digestion. mSystems 3:e00215–e00217 30073202 10.1128/mSystems.00215-17 PMC6068829

[CR15] Berry DB, Gasch AP (2008) Stress-activated genomic expression changes serve a preparative role for impending stress in yeast. Mol Biol Cell 19:4580–4587 18753408 10.1091/mbc.E07-07-0680 PMC2575158

[CR16] Berry DB, Guan Q, Hose J, Haroon S, Gebbia M, Heisler LE, Nislow C, Giaever G, Gasch AP (2011) Multiple means to the same end: the genetic basis of acquired stress resistance in yeast. PLoS Genet 7:e1002353 22102822 10.1371/journal.pgen.1002353 PMC3213159

[CR17] Bolger AM, Lohse M, Usadel B (2014) Trimmomatic: a flexible trimmer for Illumina sequence data. Bioinformatics 30:2114–2120 24695404 10.1093/bioinformatics/btu170 PMC4103590

[CR18] Bosio MC, Fermi B, Dieci G (2017a) Transcriptional control of yeast ribosome biogenesis: a multifaceted role for general regulatory factors. Transcription 8:254–260 28448767 10.1080/21541264.2017.1317378 PMC5574525

[CR19] Bosio MC, Fermi B, Spagnoli G, Levati E, Rubbi L, Ferrari R, Pellegrini M, Dieci G (2017b) Abf1 and other general regulatory factors control ribosome biogenesis gene expression in budding yeast. Nucleic Acids Res 45:4493–4506 28158860 10.1093/nar/gkx058 PMC5416754

[CR20] Boutte CC, Crosson S (2013) Bacterial lifestyle shapes stringent response activation. Trends Microbiol 21:174–180 23419217 10.1016/j.tim.2013.01.002 PMC4238387

[CR21] Boy-Marcotte E, Perrot M, Bussereau F, Boucherie H, Jacquet M (1998) Msn2p and Msn4p control a large number of genes induced at the diauxic transition which are repressed by cyclic AMP in Saccharomyces cerevisiae. J Bacteriol 180:1044–1052 9495741 10.1128/jb.180.5.1044-1052.1998 PMC106990

[CR22] Brauer MJ, Huttenhower C, Airoldi EM, Rosenstein R, Matese JC, Gresham D, Boer VM, Troyanskaya OG, Botstein D (2008) Coordination of growth rate, cell cycle, stress response, and metabolic activity in yeast. Mol Biol Cell 19:352–367 17959824 10.1091/mbc.E07-08-0779 PMC2174172

[CR23] Brodsky S, Jana T, Mittelman K, Chapal M, Kumar DK, Carmi M, Barkai N (2020) Intrinsically disordered regions direct transcription factor in vivo binding specificity. Mol Cell 79:459–471.e4 32553192 10.1016/j.molcel.2020.05.032

[CR24] Brown AJP, Budge S, Kaloriti D, Tillmann A, Jacobsen MD, Yin Z, Ene IV, Bohovych I, Sandai D, Kastora S et al (2014) Stress adaptation in a pathogenic fungus. J Exp Biol 217:144–155 24353214 10.1242/jeb.088930 PMC3867497

[CR25] Bucci V, Tzen B, Li N, Simmons M, Tanoue T, Bogart E, Deng L, Yeliseyev V, Delaney ML, Liu Q et al (2016) MDSINE: Microbial dynamical systems inference engine for microbiome time-series analyses. Genome Biol 17:121 27259475 10.1186/s13059-016-0980-6 PMC4893271

[CR26] Castrillo JI, Zeef LA, Hoyle DC, Zhang N, Hayes A, Gardner DC, Cornell MJ, Petty J, Hakes L, Wardleworth L et al (2007) Growth control of the eukaryote cell: a systems biology study in yeast. J Biol 6:4 17439666 10.1186/jbiol54 PMC2373899

[CR27] Causton HC, Ren B, Koh SS, Harbison CT, Kanin E, Jennings EG, Lee TI, True HL, Lander ES, Young RA (2001) Remodeling of yeast genome expression in response to environmental changes. Mol Biol Cell 12:323–337 11179418 10.1091/mbc.12.2.323 PMC30946

[CR28] Chasman D, Ho Y, Berry DB, Nemec CM, MacGilvray ME, Hose J, Merrill AE, Lee MV, Will JL, Coon JJ et al (2014) Pathway connectivity and signaling coordination in the yeast stress-activated signaling network. Mol Syst Biol 10:759 25411400 10.15252/msb.20145120 PMC4299600

[CR29] Cheng Z, Brar GA (2019) Global translation inhibition yields condition-dependent de-repression of ribosome biogenesis mRNAs. Nucleic Acids Res 47:5061–5073 30937450 10.1093/nar/gkz231 PMC6547411

[CR30] Costa-Mattioli M, Walter P (2020) The integrated stress response: from mechanism to disease. Science 368:eaat5314 32327570 10.1126/science.aat5314 PMC8997189

[CR31] Dai X, Zhu M, Warren M, Balakrishnan R, Okano H, Williamson JR, Fredrick K, Hwa T (2018) Slowdown of translational elongation in *Escherichia coli* under hyperosmotic stress. mBio 9:1–9 10.1128/mBio.02375-17 PMC5821080 29440576

[CR32] Davis JD, Olivença DV, Brown SP, Voit EO (2022) Methods of quantifying interactions among populations using Lotka-Volterra models. Front Syst Biol 2:1021897

[CR33] De Wever V, Reiter W, Ballarini A, Ammerer G, Brocard C (2005) A dual role for PP1 in shaping the Msn2-dependent transcriptional response to glucose starvation. EMBO J 24:4115–4123 16281053 10.1038/sj.emboj.7600871 PMC1356311

[CR34] Dever TE, Ivanov IP, Hinnebusch AG (2023) Translational regulation by uORFs and start codon selection stringency. Genes Dev 37:474–489 37433636 10.1101/gad.350752.123 PMC10393191

[CR35] Dimas Martins A, Gjini E (2020) Modeling competitive mixtures with the Lotka-Volterra framework for more complex fitness assessment between strains. Front Microbiol 11:572487 33072034 10.3389/fmicb.2020.572487 PMC7536265

[CR36] Eisen MB, Spellman PT, Brown PO, Botstein D (1998) Cluster analysis and display of genome-wide expression patterns. Proc Natl Acad Sci USA 95:14863–14868 9843981 10.1073/pnas.95.25.14863 PMC24541

[CR37] Elfving N, Chereji RV, Bharatula V, Björklund S, Morozov AV, Broach JR (2014) A dynamic interplay of nucleosome and Msn2 binding regulates kinetics of gene activation and repression following stress. Nucleic Acids Res 42:5468–5482 24598258 10.1093/nar/gku176 PMC4027177

[CR38] Estruch F, Carlson M (1993) Two homologous zinc finger genes identified by multicopy suppression in a SNF1 protein kinase mutant of *Saccharomyces cerevisiae*. Mol Cell Biol 13:3872–3881 8321194 10.1128/mcb.13.7.3872-3881.1993 PMC359918

[CR39] Garreau H, Hasan RN, Renault G, Estruch F, Boy-Marcotte E, Jacquet M (2000) Hyperphosphorylation of Msn2p and Msn4p in response to heat shock and the diauxic shift is inhibited by cAMP in *Saccharomyces cerevisiae*. Microbiol Read Engl 146:2113–2120 10.1099/00221287-146-9-2113 10974099

[CR40] Gasch AP (2002b) Yeast genomic expression studies using DNA microarrays. Methods Enzymol 350:393–414 12073326 10.1016/s0076-6879(02)50976-9

[CR41] Gasch AP (2007) Comparative genomics of the environmental stress response in ascomycete fungi. Yeast 24:961–976 17605132 10.1002/yea.1512

[CR42] Gasch AP (2002a) The environmental stress response: a common yeast response to diverse environmental stresses. In: Hohmann S, Mager WH (eds) Yeast stress responses. Springer Berlin Heidelberg, Berlin, Heidelberg, pp 11–70

[CR43] Gasch AP, Spellman PT, Kao CM, Carmel-Harel O, Eisen MB, Storz G, Botstein D, Brown PO (2000) Genomic expression programs in the response of yeast cells to environmental changes. Mol Biol Cell 11:4241–4257 11102521 10.1091/mbc.11.12.4241 PMC15070

[CR44] Gasch AP, Yu FB, Hose J, Escalante LE, Place M, Bacher R, Kanbar J, Ciobanu D, Sandor L, Grigoriev IV et al (2017) Single-cell RNA sequencing reveals intrinsic and extrinsic regulatory heterogeneity in yeast responding to stress. PLoS Biol 15:e2004050 29240790 10.1371/journal.pbio.2004050 PMC5746276

[CR45] Goddard MR, Greig D (2015) *Saccharomyces cerevisiae*: a nomadic yeast with no niche? FEMS Yeast Res 15:fov009 25725024 10.1093/femsyr/fov009 PMC4444983

[CR46] González A, Hall MN (2017) Nutrient sensing and TOR signaling in yeast and mammals. EMBO J 36:397–408 28096180 10.15252/embj.201696010 PMC5694944

[CR47] González A, Ruiz A, Casamayor A, Ariño J (2009) Normal function of the yeast TOR pathway requires the type 2C protein phosphatase Ptc1. Mol Cell Biol 29:2876–2888 19273591 10.1128/MCB.01740-08 PMC2682041

[CR48] Görner W, Durchschlag E, Martinez-Pastor MT, Estruch F, Ammerer G, Hamilton B, Ruis H, Schüller C (1998) Nuclear localization of the C2H2 zinc finger protein Msn2p is regulated by stress and protein kinase A activity. Genes Dev 12:586–597 10.1101/gad.12.4.586 PMC316529 9472026

[CR49] Gourse RL, Chen AY, Gopalkrishnan S, Sanchez-Vazquez P, Myers A, Ross W (2018) Transcriptional responses to ppGpp and DksA. Annu Rev Microbiol 72:163–184 30200857 10.1146/annurev-micro-090817-062444 PMC6586590

[CR50] Guan Q, Haroon S, Bravo DG, Will JL, Gasch AP (2012) Cellular memory of acquired stress resistance in *Saccharomyces cerevisiae*. Genetics 192:495–505 22851651 10.1534/genetics.112.143016 PMC3454879

[CR51] Hansen AS, O’Shea EK (2013) Promoter decoding of transcription factor dynamics involves a trade-off between noise and control of gene expression. Mol Syst Biol 9:704 24189399 10.1038/msb.2013.56 PMC4039373

[CR52] Hansen AS, O’Shea EK (2015a) Limits on information transduction through amplitude and frequency regulation of transcription factor activity. eLife 4:e06559 25985085 10.7554/eLife.06559 PMC4468373

[CR53] Hansen AS, O’Shea EK (2015b) Cis determinants of promoter threshold and activation timescale. Cell Rep 12:1266–1233 10.1016/j.celrep.2015.07.035 26279577

[CR54] Hansen AS, O’Shea EK (2016) Encoding four gene expression programs in the activation dynamics of a single transcription factor. Curr Biol 26:R269–R271 27046808 10.1016/j.cub.2016.02.058

[CR55] Hansen AS, Zechner C (2021) Promoters adopt distinct dynamic manifestations depending on transcription factor context. Mol Syst Biol 17:e9821 33595925 10.15252/msb.20209821 PMC7888307

[CR56] Hao N, O’Shea EK (2012) Signal-dependent dynamics of transcription factor translocation controls gene expression. Nat Struct Mol Biol 19:31–39 10.1038/nsmb.2192 PMC3936303 22179789

[CR57] Harding HP, Zhang Y, Zeng H, Novoa I, Lu PD, Calfon M, Sadri N, Yun C, Popko B, Paules R et al (2003) An integrated stress response regulates amino acid metabolism and resistance to oxidative stress. Mol Cell 11:619–633 12667446 10.1016/s1097-2765(03)00105-9

[CR58] Ho Y-H, Shishkova E, Hose J, Coon JJ, Gasch AP (2018) Decoupling yeast cell division and stress defense implicates mRNA repression in translational reallocation during stress. Curr Biol CB 28:2673–2680.e4 30078561 10.1016/j.cub.2018.06.044 PMC6132260

[CR59] Houston R, Sekine S, Sekine Y (2020) The coupling of translational control and stress responses. J Biochem 168:93–102 32484875 10.1093/jb/mvaa061

[CR60] Hsu S-B, Zhao X-Q (2012) A Lotka–Volterra competition model with seasonal succession. J Math Biol 64:109–130 21318415 10.1007/s00285-011-0408-6

[CR61] Huber A, French SL, Tekotte H, Yerlikaya S, Stahl M, Perepelkina MP, Tyers M, Rougemont J, Beyer AL, Loewith R (2011) Sch9 regulates ribosome biogenesis via Stb3, Dot6 and Tod6 and the histone deacetylase complex RPD3L. EMBO J 30:3052–3064 21730963 10.1038/emboj.2011.221 PMC3160192

[CR62] Huebert DJ, Kuan P-F, Keleş S, Gasch AP (2012) Dynamic changes in nucleosome occupancy are not predictive of gene expression dynamics but are linked to transcription and chromatin regulators. Mol Cell Biol 32:1645–1653 22354995 10.1128/MCB.06170-11 PMC3347246

[CR63] Irving SE, Choudhury NR, Corrigan RM (2021) The stringent response and physiological roles of (pp)pGpp in bacteria. Nat Rev Microbiol 19:256–271 33149273 10.1038/s41579-020-00470-y

[CR64] Jacquet M, Renault G, Lallet S, De Mey J, Goldbeter A (2003) Oscillatory nucleocytoplasmic shuttling of the general stress response transcriptional activators Msn2 and Msn4 in *Saccharomyces cerevisiae*. J Cell Biol 161:497–505 12732613 10.1083/jcb.200303030 PMC2172953

[CR65] Jorgensen P, Rupes I, Sharom JR, Schneper L, Broach JR, Tyers M (2004) A dynamic transcriptional network communicates growth potential to ribosome synthesis and critical cell size. Genes Dev 18:2491–2505 15466158 10.1101/gad.1228804 PMC529537

[CR66] Jouhten P, Ponomarova O, Gonzalez R, Patil KR (2016) *Saccharomyces cerevisiae* metabolism in ecological context. FEMS Yeast Res 16:fow080 27634775 10.1093/femsyr/fow080 PMC5050001

[CR67] Kocik RA, Gasch AP (2022) Breadth and specificity in pleiotropic protein kinase A activity and environmental responses. Front Cell Dev Biol 10:803392 35252178 10.3389/fcell.2022.803392 PMC8888911

[CR68] Kolberg L, Raudvere U, Kuzmin I, Adler P, Vilo J, Peterson H (2023) g:Profiler—interoperable web service for functional enrichment analysis and gene identifier mapping (2023 update). Nucleic Acids Res 51:W207–W212 37144459 10.1093/nar/gkad347 PMC10320099

[CR69] Korem Kohanim Y, Levi D, Jona G, Towbin BD, Bren A, Alon U (2018) A bacterial growth law out of steady state. Cell Rep 23:2891–2900 29874577 10.1016/j.celrep.2018.05.007

[CR70] Kuang Z, Pinglay S, Ji H, Boeke JD (2017) Msn2/4 regulate expression of glycolytic enzymes and control transition from quiescence to growth. eLife 6:e29938 28949295 10.7554/eLife.29938 PMC5634782

[CR71] Kunkel J, Luo X, Capaldi AP (2019) Integrated TORC1 and PKA signaling control the temporal activation of glucose-induced gene expression in yeast. Nat Commun 10:1–11 31395866 10.1038/s41467-019-11540-y PMC6687784

[CR72] Kusama K, Suzuki Y, Kurita E, Kawarasaki T, Obara K, Okumura F, Kamura T, Nakatsukasa K (2022) Dot6/Tod6 degradation fine-tunes the repression of ribosome biogenesis under nutrient-limited conditions. iScience 25:103986 35310337 10.1016/j.isci.2022.103986 PMC8924686

[CR73] Langmead B, Salzberg SL (2012) Fast gapped-read alignment with Bowtie 2. Nat Methods 9:357–359 22388286 10.1038/nmeth.1923 PMC3322381

[CR74] Lee MV, Topper SE, Hubler SL, Hose J, Wenger CD, Coon JJ, Gasch AP (2011) A dynamic model of proteome changes reveals new roles for transcript alteration in yeast. Mol Syst Biol 7:514 21772262 10.1038/msb.2011.48 PMC3159980

[CR75] Lenssen E, James N, Pedruzzi I, Dubouloz F, Cameroni E, Bisig R, Maillet L, Werner M, Roosen J, Petrovic K et al (2005) The Ccr4-Not complex independently controls both Msn2-dependent transcriptional activation—via a newly identified Glc7/Bud14 type I protein phosphatase module-and TFIID promoter distribution. Mol Cell Biol 25:488–498 15601868 10.1128/MCB.25.1.488-498.2005 PMC538800

[CR76] Levy SF, Ziv N, Siegal ML (2012) Bet hedging in yeast by heterogeneous, age-correlated expression of a stress protectant. PLoS Biol 10:e1001325 22589700 10.1371/journal.pbio.1001325 PMC3348152

[CR77] Li S, Giardina DM, Siegal ML (2018) Control of nongenetic heterogeneity in growth rate and stress tolerance of *Saccharomyces cerevisiae* by cyclic AMP-regulated transcription factors. PLoS Genet 14:e1007744 30388117 10.1371/journal.pgen.1007744 PMC6241136

[CR78] Liang J, Tang H, Snyder LF, Youngstrom CE, He BZ (2023) Divergence of TORC1-mediated stress response leads to novel acquired stress resistance in a pathogenic yeast. PLoS Pathog 19:e1011748 37871123 10.1371/journal.ppat.1011748 PMC10621968

[CR79] Liko D, Conway MK, Grunwald DS, Heideman W (2010) Stb3 plays a role in the glucose-induced transition from quiescence to growth in *Saccharomyces cerevisiae*. Genetics 185:797–810 20385783 10.1534/genetics.110.116665 PMC2907202

[CR80] Liko D, Slattery MG, Heideman W (2007) Stb3 binds to ribosomal RNA processing element motifs that control transcriptional responses to growth in *Saccharomyces cerevisiae*. J Biol Chem 282:26623–26628 17616518 10.1074/jbc.M704762200

[CR81] Lippman SI, Broach JR (2009) Protein kinase A and TORC1 activate genes for ribosomal biogenesis by inactivating repressors encoded by Dot6 and its homolog Tod6. Proc Natl Acad Sci USA 106:19928–19933 10.1073/pnas.0907027106 PMC2775034 19901341

[CR82] Lu C, Brauer MJ, Botstein D (2009) Slow growth induces heat-shock resistance in normal and respiratory-deficient yeast. Mol Biol Cell 20:891–903 19056679 10.1091/mbc.E08-08-0852 PMC2633392

[CR83] Mahendrawada L, Warfield L, Donczew R, Hahn S (2025) Low overlap of transcription factor DNA binding and regulatory targets. Nature 642:796–804 40240607 10.1038/s41586-025-08916-0 PMC12882699

[CR84] Marion RM, Regev A, Segal E, Barash Y, Koller D, Friedman N, O’Shea EK (2004) Sfp1 is a stress- and nutrient-sensitive regulator of ribosomal protein gene expression. Proc Natl Acad Sci USA 101:14315–14322 15353587 10.1073/pnas.0405353101 PMC521938

[CR85] Martínez-Pastor MT, Marchler G, Schüller C, Marchler-Bauer A, Ruis H, Estruch F (1996) The *Saccharomyces cerevisiae* zinc finger proteins Msn2p and Msn4p are required for transcriptional induction through the stress-response element (STRE). EMBO J 15:2227–2235 8641288 PMC450147

[CR86] Mindel V, Brodsky S, Cohen A, Manadre W, Jonas F, Carmi M, Barkai N (2024) Intrinsically disordered regions of the Msn2 transcription factor encode multiple functions using interwoven sequence grammars. Nucleic Acids Res 52:2260–2272 38109289 10.1093/nar/gkad1191 PMC10954448

[CR87] Ni L, Bruce C, Hart C, Leigh-Bell J, Gelperin D, Umansky L, Gerstein MB, Snyder M (2009) Dynamic and complex transcription factor binding during an inducible response in yeast. Genes Dev 23:1351–1363 19487574 10.1101/gad.1781909 PMC2701586

[CR88] O’Duibhir E, Lijnzaad P, Benschop JJ, Lenstra TL, van Leenen D, Groot Koerkamp MJ, Margaritis T, Brok MO, Kemmeren P, Holstege FC (2014) Cell cycle population effects in perturbation studies. Mol Syst Biol 10:732 24952590 10.15252/msb.20145172 PMC4265054

[CR89] Pontes MH, Groisman EA (2019) Slow growth determines nonheritable antibiotic resistance in *Salmonella enterica*. Sci Signal 12:eaax3938 31363068 10.1126/scisignal.aax3938 PMC7206539

[CR90] Purvis JE, Karhohs KW, Mock C, Batchelor E, Loewer A, Lahav G (2012) p53 dynamics control cell fate. Science 336:1440–1444 22700930 10.1126/science.1218351 PMC4162876

[CR91] Purvis JE, Lahav G (2013) Encoding and decoding cellular information through signaling dynamics. Cell 152:945–956 23452846 10.1016/j.cell.2013.02.005 PMC3707615

[CR92] Regenberg B, Grotkjaer T, Winther O, Fausbøll A, Akesson M, Bro C, Hansen LK, Brunak S, Nielsen J (2006) Growth-rate regulated genes have profound impact on interpretation of transcriptome profiling in *Saccharomyces cerevisiae*. Genome Biol 7:R107 17105650 10.1186/gb-2006-7-11-r107 PMC1794586

[CR93] Rep M, Reiser V, Gartner U, Thevelein JM, Hohmann S, Ammerer G, Ruis H (1999) Osmotic stress-induced gene expression in *Saccharomyces cerevisiae* requires Msn1p and the novel nuclear factor Hot1p. Mol Cell Biol 19:5474–5485 10409737 10.1128/mcb.19.8.5474 PMC84389

[CR94] Robinson MD, Oshlack A (2010) A scaling normalization method for differential expression analysis of RNA-seq data. Genome Biol 11:R25 20196867 10.1186/gb-2010-11-3-r25 PMC2864565

[CR95] Saldanha AJ (2004) Java Treeview-extensible visualization of microarray data. Bioinformatics 20:3246–3248 15180930 10.1093/bioinformatics/bth349

[CR96] Santana-Garcia W, Castro-Mondragon JA, Padilla-Gálvez M, Nguyen NTT, Elizondo-Salas A, Ksouri N, Gerbes F, Thieffry D, Vincens P, Contreras-Moreira B et al (2022) RSAT 2022: regulatory sequence analysis tools. Nucleic Acids Res 50:W670–W676 35544234 10.1093/nar/gkac312 PMC9252783

[CR97] Santhanam A, Hartley A, Düvel K, Broach JR, Garrett S (2004) PP2A phosphatase activity is required for stress and Tor kinase regulation of yeast stress response factor Msn2p. Eukaryot Cell 3:1261–1271 15470255 10.1128/EC.3.5.1261-1271.2004 PMC522594

[CR98] Schawalder SB, Kabani M, Howald I, Choudhury U, Werner M, Shore D (2004) Growth-regulated recruitment of the essential yeast ribosomal protein gene activator Ifh1. Nature 432:1058–1061 15616569 10.1038/nature03200

[CR99] Schuetz R, Zamboni N, Zampieri M, Heinemann M, Sauer U (2012) Multidimensional optimality of microbial metabolism. Science 336:601–604 22556256 10.1126/science.1216882

[CR100] Scott M, Hwa T (2011) Bacterial growth laws and their applications. Curr Opin Biotechnol 22:559–565 21592775 10.1016/j.copbio.2011.04.014 PMC3152618

[CR101] Scott M, Hwa T (2023) Shaping bacterial gene expression by physiological and proteome allocation constraints. Nat Rev Microbiol 21:327–342 36376406 10.1038/s41579-022-00818-6 PMC10121745

[CR102] Shore D, Nasmyth K (1987) Purification and cloning of a DNA binding protein from yeast that binds to both silencer and activator elements. Cell 51:721–732 3315231 10.1016/0092-8674(87)90095-x

[CR103] Simpson-Lavy K, Kupiec M (2019) Carbon catabolite repression: not only for glucose. Curr Genet 65:1321–1323 31119370 10.1007/s00294-019-00996-6

[CR104] Smith A, Ward MP, Garrett S (1998) Yeast PKA represses Msn2p / Msn4p-dependent gene expression to regulate growth, stress response and glycogen accumulation. EMBO J 17:3556–3564 9649426 10.1093/emboj/17.13.3556 PMC1170692

[CR105] Stein RR, Bucci V, Toussaint NC, Buffie CG, Rätsch G, Pamer EG, Sander C, Xavier JB (2013) Ecological modeling from time-series inference: insight into dynamics and stability of intestinal microbiota. PLoS Comput Biol 9:e1003388 24348232 10.1371/journal.pcbi.1003388 PMC3861043

[CR106] Stewart-Ornstein J, Nelson C, DeRisi J, Weissman JS, El-Samad H (2013) Msn2 coordinates a stoichiometric gene expression program. Curr Biol 23:2336–2345 24210615 10.1016/j.cub.2013.09.043 PMC4072881

[CR107] Sweeney K, McClean MN (2023) Transcription factor localization dynamics and DNA binding drive distinct promoter interpretations. Cell Rep 42:112426 37087734 10.1016/j.celrep.2023.112426 PMC10292158

[CR108] Teixeira MC, Viana R, Palma M, Oliveira J, Galocha M, Mota MN, Couceiro D, Pereira MG, Antunes M, Costa IV et al (2023) YEASTRACT+: a portal for the exploitation of global transcription regulation and metabolic model data in yeast biotechnology and pathogenesis. Nucleic Acids Res 51:D785–D791 36350610 10.1093/nar/gkac1041 PMC9825512

[CR109] Turcotte B, Liang XB, Robert F, Soontorngun N (2010) Transcriptional regulation of nonfermentable carbon utilization in budding yeast. FEMS Yeast Res 10:2–13 19686338 10.1111/j.1567-1364.2009.00555.x PMC5003605

[CR110] Vermeersch L, Cool L, Gorkovskiy A, Voordeckers K, Wenseleers T, Verstrepen KJ (2022) Do microbes have a memory? History-dependent behavior in the adaptation to variable environments. Front Microbiol 13:1004488 36299722 10.3389/fmicb.2022.1004488 PMC9589428

[CR111] Warner JR (1999) The economics of ribosome biosynthesis in yeast. Trends Biochem Sci 24:437–440 10542411 10.1016/s0968-0004(99)01460-7

[CR112] Wu C, Balakrishnan R, Braniff N, Mori M, Manzanarez G, Zhang Z, Hwa T (2022) Cellular perception of growth rate and the mechanistic origin of bacterial growth law. Proc Natl Acad Sci USA 119:1–9 10.1073/pnas.2201585119 PMC9171811 35544692

[CR113] Zakrzewska A, van Eikenhorst G, Burggraaff JEC, Vis DJ, Hoefsloot H, Delneri D, Oliver SG, Brul S, Smits GJ (2011) Genome-wide analysis of yeast stress survival and tolerance acquisition to analyze the central trade-off between growth rate and cellular robustness. Mol Biol Cell 22:4435–4446 21965291 10.1091/mbc.E10-08-0721 PMC3216668

[CR114] Zhang H, Zhao Y, Zhu J-K (2020) Thriving under stress: how plants balance growth and the stress response. Dev Cell 55:529–543 33290694 10.1016/j.devcel.2020.10.012

[CR115] Zhu C, Byers KJRP, McCord RP, Shi Z, Berger MF, Newburger DE, Saulrieta K, Smith Z, Shah MV, Radhakrishnan M et al (2009) High-resolution DNA-binding specificity analysis of yeast transcription factors. Genome Res 19:556–566 19158363 10.1101/gr.090233.108 PMC2665775

[CR116] Zhu M, Dai X (2023) Stringent response ensures the timely adaptation of bacterial growth to nutrient downshift. Nat Commun 14:467 36709335 10.1038/s41467-023-36254-0 PMC9884231

[CR117] Zhu M, Mu H, Dai X (2024) Integrated control of bacterial growth and stress response by (p)ppGpp in *Escherichia coli*: a seesaw fashion. iScience 27:108818 38299113 10.1016/j.isci.2024.108818 PMC10828813

[CR118] Zhu M, Pan Y, Dai X (2019) p)ppGpp: the magic governor of bacterial growth economy. Curr Genet 65:1121–1125 30993414 10.1007/s00294-019-00973-z

