# Supplementary material for: The importance of regulated resource reallocation during dynamic environmental shifts in yeast
Source: EMBO J. 2026 Mar 11;45(8):2808–30. doi: 10.1038/s44318-026-00727-x (PMC13084002; doi:10.1038/s44318-026-00727-x)
Supplement: Supplementary file 6 — Source data Fig. 1 [file 44318_2026_727_MOESM6_ESM.zip › Figure 1/Figure_1C/Fig1C_README.docx]

Fig 1C README:

Top panel: growth rate k calculated for each replicate of each denoted strain.

Bottom panel: growth rates relative to the wild-type strain as shown in the figure. Average and standard deviation shown in the figure are included.
