# Supplementary material for: The importance of regulated resource reallocation during dynamic environmental shifts in yeast
Source: EMBO J. 2026 Mar 11;45(8):2808–30. doi: 10.1038/s44318-026-00727-x (PMC13084002; doi:10.1038/s44318-026-00727-x)
Supplement: Supplementary file 6 — Source data Fig. 1 [file 44318_2026_727_MOESM6_ESM.zip › Figure 1/Figure_1D/Fig1D_README.docx]

Fig 1D README:

Data list the growth rate k calculated for each replicate of each denoted strain. Average and standard deviation shown in the figure are included.
