# Supplementary material for: The importance of regulated resource reallocation during dynamic environmental shifts in yeast
Source: EMBO J. 2026 Mar 11;45(8):2808–30. doi: 10.1038/s44318-026-00727-x (PMC13084002; doi:10.1038/s44318-026-00727-x)
Supplement: Supplementary file 7 — Source data Fig. 2 [file 44318_2026_727_MOESM7_ESM.zip › Figure 2/Figure_2B-EV1/Fig2B-EV1_README.docx]

Figure 2B and EV1 – README

Data represent acquired stress scores for deonted strains as outlined in the Methods. Fig 2B shows a subset of the data shown in Figure_EV1.

| Column headers: | Rep # | Paired replicates (WT and one mutant) | | |  |
| --- | --- | --- | --- | --- | --- |
|  | Strain | AGY strain # |  |  |  |
|  | Strain Name | WT or name of mutant strain | | |  |
|  | Timepoint | Time following addition of NaCl (T0 = before salt, T10 = 10 minutes post-salt, etc.) | | | |
|  | H2O2 Doses (mM) 1-20 | Score 0-3 (see methods), corresponding to growth of strain at Timepoint and H2O2 Dose | | | |
|  | Sum of Scores | Sum of row of H2O2 Doses (mM) 1-20 for each Timepoint | | | |
