# Supplementary material for: The importance of regulated resource reallocation during dynamic environmental shifts in yeast
Source: EMBO J. 2026 Mar 11;45(8):2808–30. doi: 10.1038/s44318-026-00727-x (PMC13084002; doi:10.1038/s44318-026-00727-x)
Supplement: Supplementary file 8 — Source data Fig. 3 [file 44318_2026_727_MOESM8_ESM.zip › Figure 3/Figure_3A-C/Fig3_README.docx]

Figure 3 - README

Data include quantified microscopy data as described in Methods. Figure 3A shows the traces in the headers whereas Figures 3B-C show box or scatter plots of specific columns as indicated in the figure legends.

| Fig 3 column headers: | Cell ID Number |
| --- | --- |
|  |  |
|  | Genotype inferred from iRFP (WT=1; ∆ mutant=0) |
|  |  |
|  | Msn2_ nuclear-cytoplasmic ratio at each timepoint |
|  | Msn2_ratio normalized at each time point as described in Methods |
