# Supplementary material for: The importance of regulated resource reallocation during dynamic environmental shifts in yeast
Source: EMBO J. 2026 Mar 11;45(8):2808–30. doi: 10.1038/s44318-026-00727-x (PMC13084002; doi:10.1038/s44318-026-00727-x)
Supplement: Supplementary file 9 — Source data Fig. 4 [file 44318_2026_727_MOESM9_ESM.zip › Figure 4/Figure_4A-D/Fig4_README.docx]

Figure 4 – README

Data include quantified microscopy data as described in Methods.

| Fig 4: | Cell ID Number |  |
| --- | --- | --- |
|  |  |  |
|  | Genotype inferred from iRFP (WT=1; ∆ mutant=0) | |
|  |  |  |
|  | Dot6_ nuclear-cytoplasmic ratio at each timepoint | |
|  | Dot6_ratio normalized at each time point as described in Methods | |
