# Supplementary material for: The importance of regulated resource reallocation during dynamic environmental shifts in yeast
Source: EMBO J. 2026 Mar 11;45(8):2808–30. doi: 10.1038/s44318-026-00727-x (PMC13084002; doi:10.1038/s44318-026-00727-x)
Supplement: Supplementary file 10 — Source data Fig. 5 [file 44318_2026_727_MOESM10_ESM.zip › Figure_5/Figure_5D/Fig5D_README.docx]

Figure 5D – README

Each column represents the number of upstream transcription-factor binding sites for genes in denoted clusters as outlined in Methods.
