# Supplementary material for: The importance of regulated resource reallocation during dynamic environmental shifts in yeast
Source: EMBO J. 2026 Mar 11;45(8):2808–30. doi: 10.1038/s44318-026-00727-x (PMC13084002; doi:10.1038/s44318-026-00727-x)
Supplement: Supplementary file 10 — Source data Fig. 5 [file 44318_2026_727_MOESM10_ESM.zip › Figure_5/Figure_5A-C/Fig5_README.docx]

Figure 5 – README

RNA-seq data as outlined in Fig 5 legend and Methods.

| Fig 5: Gene sorted in order shown in Fig 5 | UID | ORF identifier |
| --- | --- | --- |
|  | NAME | Gene name and annotation |
|  | Yeastract_DirectBinding_Msn2-Msn4 | yes = promoter bound by Msn2/4 in Yeastract |
|  | Huebert_DirectPromoterBinding_Msn2 | yes = promoter bound by Msn2/4 in Huebert et al. |
|  | log2 # yeastract TF regulators | Total # of transcription factor (TF) regulators reported in Yeastract |
|  | # STRE within -500bp | # of STRE within 500 bp upstream |
|  | GATGAG within -500 bp | # GATGAG within 500 bp upstream |
|  | DE in dot6tod6 mutant (FDR < 0.05 at at least 2 time points) | Scored as differentially expressed (DE) in dot6tod6 |
|  | DE in msn2msn4 mutant (FDR < 0.05 at at least 2 time points) | Scored as differentially expressed (DE) in msn2msn4 |
|  | Remaining columns | log2(fold-difference) in expression between denoted strains |
