# Supplementary material for: The importance of regulated resource reallocation during dynamic environmental shifts in yeast
Source: EMBO J. 2026 Mar 11;45(8):2808–30. doi: 10.1038/s44318-026-00727-x (PMC13084002; doi:10.1038/s44318-026-00727-x)
Supplement: Supplementary file 11 — Source data Fig. 7 [file 44318_2026_727_MOESM11_ESM.zip › Figure_7/Fig7_README.docx]

Figure 7 README

This R script outputs the phase map shown in Figure 7 representing competition outcomes between wild type and *msn2∆msn4∆* yeast with the y axis corresponding to the severity (death toll) and the x axis corresponding to the frequency (time between normalized by log phase doubling rate) of secondary stresses.
