# Supplementary material for: The importance of regulated resource reallocation during dynamic environmental shifts in yeast
Source: EMBO J. 2026 Mar 11;45(8):2808–30. doi: 10.1038/s44318-026-00727-x (PMC13084002; doi:10.1038/s44318-026-00727-x)
Supplement: Supplementary file 12 — Figure EV1 Source Data [file 44318_2026_727_MOESM12_ESM.zip › Figure_EV1/Fig2B-EV1A-D_README.docx]

Figure 2B and EV1 – README

| Fig 2 and EV1 : | Rep # | Paired replicates (WT and one mutant) | | |  |
| --- | --- | --- | --- | --- | --- |
|  | Strain | AGY strain # |  |  |  |
|  | Strain Name | WT or name of mutant strain | | |  |
|  | Timepoint | Time following addition of NaCl (T0 = before salt, T10 = 10 minutes post-salt, etc.) | | | |
|  | H2O2 Doses (mM) 1-20 | Score 0-3 (see methods), corresponding to growth of strain at Timepoint and H2O2 Dose | | | |
|  | Sum of Scores | Sum of row of H2O2 Doses (mM) 1-20 for each Timepoint | | | |

Figure 2 plot is a subset of the data shown in Figure EV1
