# Supplementary material for: The importance of regulated resource reallocation during dynamic environmental shifts in yeast
Source: EMBO J. 2026 Mar 11;45(8):2808–30. doi: 10.1038/s44318-026-00727-x (PMC13084002; doi:10.1038/s44318-026-00727-x)
Supplement: Supplementary file 13 — Figure EV2 Source Data [file 44318_2026_727_MOESM13_ESM.zip › Figure_EV2/FigEV2_README.docx]

Figure EV2 – README

| **WT Dot6-GFP & WT Tod6-GFP** |  |  |  |  |  |  |  |  |  |  |  |  |  |  |  |  |  |  |
| --- | --- | --- | --- | --- | --- | --- | --- | --- | --- | --- | --- | --- | --- | --- | --- | --- | --- | --- |
| Each row represents data for an individual cell from strain AGY2247 or AGY2233. | | | | |  |  |  |  |  |  |  |  |  |  |  |  |  |  |
| There are 40 time point measurements, labeled T01 through T40. | | | |  |  |  |  |  |  |  |  |  |  |  |  |  |  |  |
| Each measurement was taken 6 minutes apart, with T01 measurement taken at the beginning of the experiment. | | | | | | | |  |  |  |  |  |  |  |  |  |  |  |
|  |  |  |  |  |  |  |  |  |  |  |  |  |  |  |  |  |  |  |
| cell_numb | unique identifier for each cell in these experiments | | | | |  |  |  |  |  |  |  |  |  |  |  |  |  |
| strain | yeast strain of that cell | | |  |  |  |  |  |  |  |  |  |  |  |  |  |  |  |
| rep | which of the 1 replicate experiment each cell comes from | | | | | |  |  |  |  |  |  |  |  |  |  |  |  |
| GFP_ratio_T01 - GFP_ratio_T40 | nuclear versus cytoplasmic ratio for Dot6 or Tod6 at each measured time point | | | | | | | |  |  |  |  |  |  |  |  |  |  |
| iRFP_ratio_T01 - iRFP_ratio_T39 | ratio of average of top 5% of pixels divided by the median pixel intensity of all pixels in the cell for iRFP at each measured time point | | | | | | | | | | | |  |  |  |  |  |  |
|  |  |  |  |  |  |  |  |  |  |  |  |  |  |  |  |  |  |  |
|  |  |  |  |  |  |  |  |  |  |  |  |  |  |  |  |  |  |  |
| **WT & Tod6-GFP msn2msn4 mutants** |  |  |  |  |  |  |  |  |  |  |  |  |  |  |  |  |  |  |
| Each row represents data for an individual cell from strain AGY2248 or AGY2260. | | | | |  |  |  |  |  |  |  |  |  |  |  |  |  |  |
| There are 39 time point measurements, labeled T01 through T39. | | | |  |  |  |  |  |  |  |  |  |  |  |  |  |  |  |
| Each measurement was taken 6 minutes apart, with T01 measurement taken at the beginning of the experiment. | | | | | | | |  |  |  |  |  |  |  |  |  |  |  |
|  |  |  |  |  |  |  |  |  |  |  |  |  |  |  |  |  |  |  |
| cell_numb | unique identifier for each cell in these experiments | | | | |  |  |  |  |  |  |  |  |  |  |  |  |  |
| strain | yeast strain of that cell | | |  |  |  |  |  |  |  |  |  |  |  |  |  |  |  |
| rep | which of the 2 replicate experiment each cell comes from | | | | | |  |  |  |  |  |  |  |  |  |  |  |  |
| Msn2_ratio_T01 - Msn2_ratio_T39 | nuclear versus cytoplasmic ratio for Msn2 at each measured time point | | | | | | |  |  |  |  |  |  |  |  |  |  |  |
| Tod6_ratio_T01 - Tod6_ratio_T39 | nuclear versus cytoplasmic ratio for Tod6 at each measured time point | | | | | | |  |  |  |  |  |  |  |  |  |  |  |
| Tod6_med_T01 - Tod6_med_T39 | median Dot6-GFP signal at each measured time point | | | | |  |  |  |  |  |  |  |  |  |  |  |  |  |
| iRFP_ratio_T01 - iRFP_ratio_T39 | ratio of average of top 5% of pixels divided by the median pixel intensity of all pixels in the cell for iRFP at each measured time point | | | | | | | | | | | |  |  |  |  |  |  |
| Tod6_AUC_prestress | area under the curve (AUC) of Tod6 nuclear signal for timepoints T01 - T12 | | | | | | |  |  |  |  |  |  |  |  |  |  |  |
| Tod6_AUC_acclimation | area under the curve (AUC) of Tod6 nuclear signal for timepoints T24 - T37 | | | | | | |  |  |  |  |  |  |  |  |  |  |  |
| Tod6_acute_peak_height | acute stress peak height of Tod6 as described in the Methods | | | | | |  |  |  |  |  |  |  |  |  |  |  |  |
|  |  |  |  |  |  |  |  |  |  |  |  |  |  |  |  |  |  |  |
|  |  |  |  |  |  |  |  |  |  |  |  |  |  |  |  |  |  |  |
|  |  |  |  |  |  |  |  |  |  |  |  |  |  |  |  |  |  |  |
|  |  |  |  |  |  |  |  |  |  |  |  |  |  |  |  |  |  |  |
|  |  |  |  |  |  |  |  |  |  |  |  |  |  |  |  |  |  |  |
|  |  |  |  |  |  |  |  |  |  |  |  |  |  |  |  |  |  |  |
|  |  |  |  |  |  |  |  |  |  |  |  |  |  |  |  |  |  |  |
|  |  |  |  |  |  |  |  |  |  |  |  |  |  |  |  |  |  |  |
|  |  |  |  |  |  |  |  |  |  |  |  |  |  |  |  |  |  |  |
|  |  |  |  |  |  |  |  |  |  |  |  |  |  |  |  |  |  |  |
