# Supplementary material for: The importance of regulated resource reallocation during dynamic environmental shifts in yeast
Source: EMBO J. 2026 Mar 11;45(8):2808–30. doi: 10.1038/s44318-026-00727-x (PMC13084002; doi:10.1038/s44318-026-00727-x)
Supplement: Supplementary file 14 — Figure EV3 Source Data [file 44318_2026_727_MOESM14_ESM.zip › Figure_EV3/FigEV3_README.docx]

Figure EV3 – README

Data include log2(fold-change) expression values for denoted strain comparisons. One of the *dot6∆tod6∆* mutant replicates shown in B) was taken from the time course for display. Boxplots show the distribution of values as described in the figure legend.
