# Supplementary material for: The importance of regulated resource reallocation during dynamic environmental shifts in yeast
Source: EMBO J. 2026 Mar 11;45(8):2808–30. doi: 10.1038/s44318-026-00727-x (PMC13084002; doi:10.1038/s44318-026-00727-x)
Supplement: Supplementary file 15 — Figure EV4 Source Data [file 44318_2026_727_MOESM15_ESM.zip › Figure_EV4/FigE4_README.docx]

Figure EV4 – README

Data include quantified microscopy data as described in Methods.

| Each row represents data for an individual cell from strain AGY1328 or AGY2255. | |
| --- | --- |
| There are 40 time point measurements, labeled T01 through T40. | |
| Each measurement was taken 6 minutes apart, with T01 measurement taken at the beginning of the experiment. | |
|  |  |
| cell_numb | unique identifier for each cell in these experiments |
| strain | yeast strain of that cell |
| rep | which of the 1 replicate experiment each cell comes from |
| Msn2_ratio_T01 - Msn2_ratio_T40 | nuclear versus cytoplasmic ratio for Msn2 at each measured time point |
| Dot6_ratio_T01 - Dot6_ratio_T40 | nuclear versus cytoplasmic ratio for Dot6 at each measured time point |
| Dot6_med_T01 - Dot6_med_T40 | median Dot6-GFP signal at each measured time point |
| iRFP_ratio_T01 - iRFP_ratio_T40 | ratio of average of top 5% of pixels divided by the median pixel intensity of all pixels in the cell for iRFP at each measured time point |
| Dot6_AUC_prestress | area under the curve (AUC) of Dot6 nuclear signal for timepoints T01 - T12 |
| Dot6_AUC_acclimation | area under the curve (AUC) of Dot6 nuclear signal for timepoints T24 - T37 |
| Dot6_acute_peak_height | acute stress peak height of Dot6 as described in the Methods |
