# Supplementary material for: The importance of regulated resource reallocation during dynamic environmental shifts in yeast
Source: EMBO J. 2026 Mar 11;45(8):2808–30. doi: 10.1038/s44318-026-00727-x (PMC13084002; doi:10.1038/s44318-026-00727-x)
Supplement: Supplementary file 16 — Expanded View Figures [file 44318_2026_727_MOESM16_ESM.pdf]

Expanded View Figures

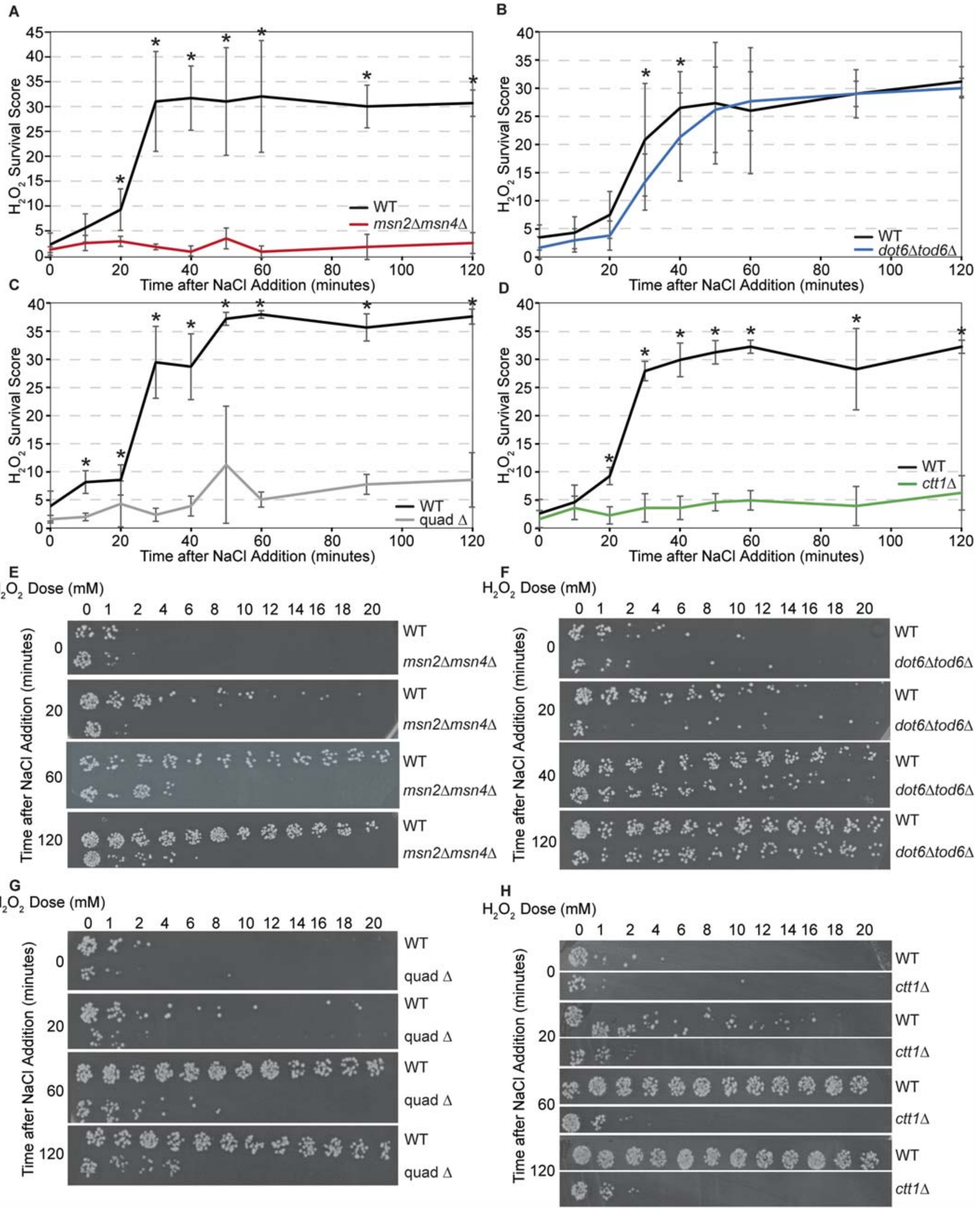

 © The Author(s)

Figure EV1. Msn2/4 and Dot6/Tod6 responses are important for acquired stress resistance.

Related to Fig. 2. (**A–D**) The average change in H_2O_2 survival scores for wild-type (black), *msn2Δmsn4Δ* (red), *dot6Δtod6Δ* (blue), quad Δ (gray), and *ctt1Δ* (green) cells +/− 1 standard deviation, as described in Fig. 2 ($n = 3$ except for (**C**) where $n = 6$). Colored lines are as shown in Fig. 2, along with the paired wild-type culture done side-by-side with each mutant. (**E–H**) Representative images of cell viability across doses of H_2O_2 and time used to calculate H_2O_2 survival scores. Paired *t* test, exact *P* values are available in Dataset EV1. Source data are available online for this figure.

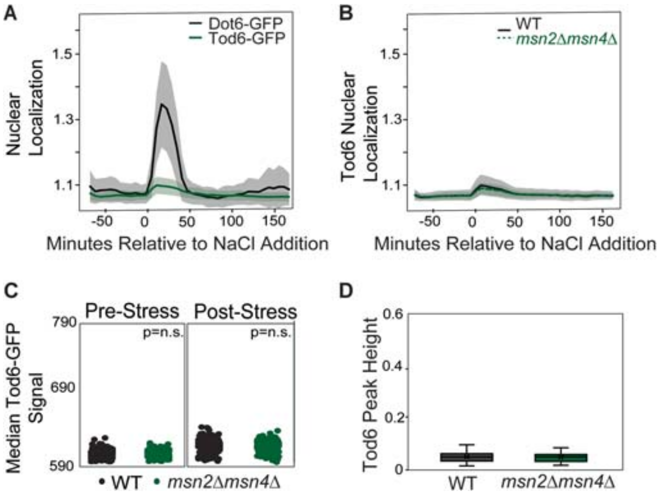

Figure EV2. Loss of *MSN2/4* leads does not affect Tod6 abundance and nuclear localization.

Y-axes are set to scales comparable to Fig. 3B–D for comparison with Dot6. (**A**) Dot6-GFP in an iRFP-marked strain was mixed with cells expressing Tod6-GFP, both expressed from the native genomic loci, and nuclear translocation of each factor was scored before and after NaCl treatment in the same device ($n = 132$ cells: WT, 56, *msn2Δmsn4Δ*, 76). The results confirm that Dot6-GFP shows strong nuclear relocalization in response to NaCl, whereas Tod6-GFP shows only a weak change under the conditions used here. (**B**) The population average of Tod6-GFP nuclear/cytoplasmic ratio in wild-type (black line) and *msn2Δmsn4Δ* (dashed green line) cells +/− one standard deviation (shading). The two strains are statistically indistinguishable ($P > 0.1$) (**C**) Distribution of median Tod6-GFP signal scored before (0–72 min) or after (120–216 min) NaCl treatment (see "Methods" for details) for WT and *msn2Δmsn4Δ* cells; $P = 0.138$ (left panel), 0.817 (right panel), Wilcoxon rank-sum test; n.s. = not significant. (**D**) Distribution of Tod6-GFP acute-stress peak heights across 132 cells, see "Methods". $P = 0.896$, Wilcoxon rank-sum test. Boxplots show median (line) and 0.25 and 0.75 quartiles (box), with whiskers extending from minimum to maximum excluding outliers (circles) that are <0.25 quartile–1.5× interquartile range or >0.75 quartile–1.5× interquartile range. This figure shows that cells lacking *MSN2/MSN4* show no difference in Tod6-GFP levels or activation compared to wild-type. Source data are available online for this figure.

© The Author(s)

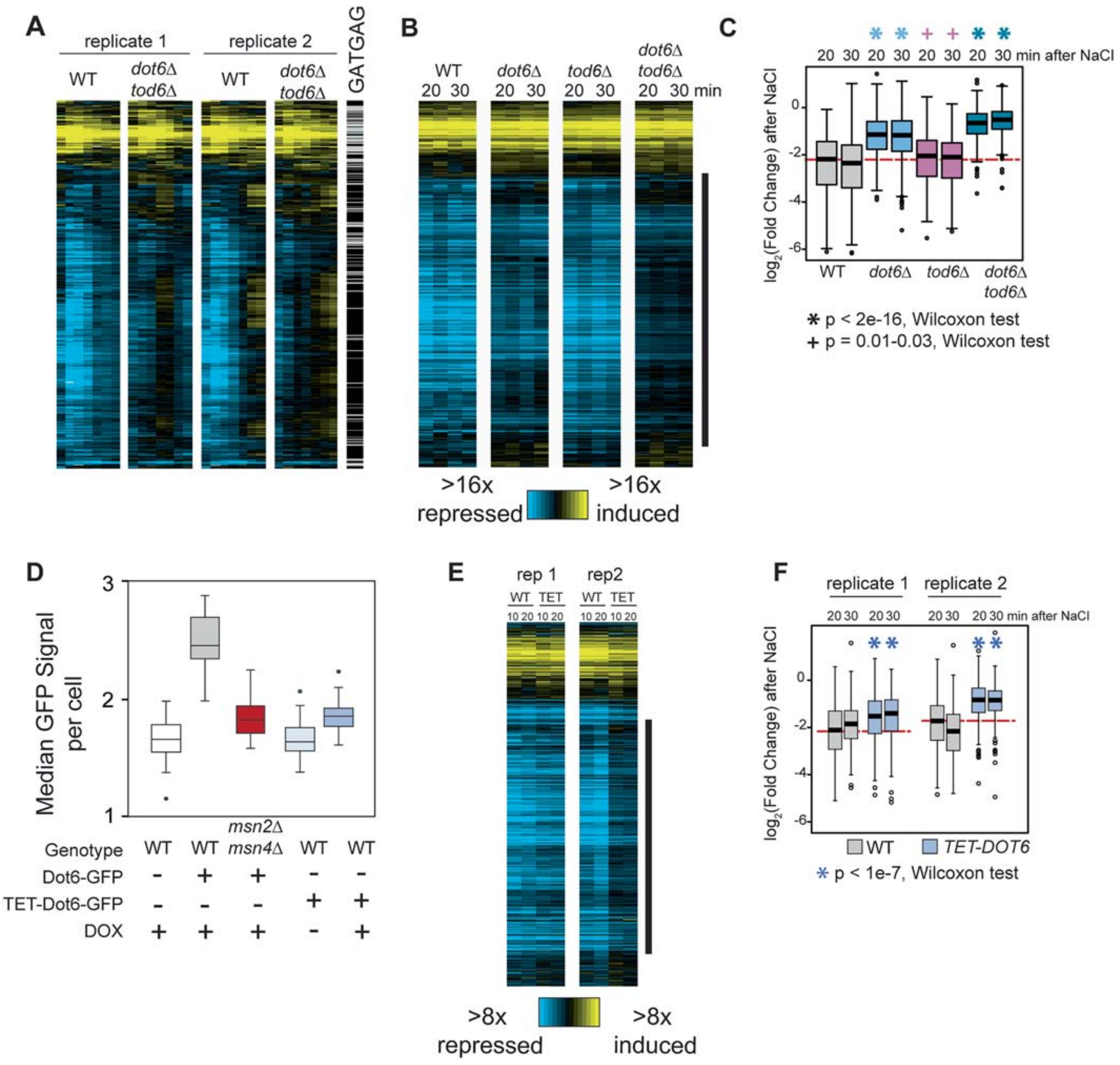

Figure EV3. Dot6 contributes to gene repression more than Tod6 during NaCl stress.

(**A**) Log_2(fold change) of 489 genes significantly affected by *dot6Δtod6Δ* deletion in the time course (10–90 min), as shown in Fig. 5A, see Methods for details. Presence of GATGAG sequences within 500 bp upstream of each gene is indicated with a black line next to each gene. (**B**) Expression of the same genes in single-gene deletion of *DOT6* or *TOD6* at the peak of the expression response (20 and 30 min). (**C**) Boxplots of replicate-averaged data from the highlighted cluster from (**B**) indicated with the black bar (379 genes). The median repression of wild-type at 20 min is indicated with a red-dashed line. Note the *dot6Δ* mutant has a partial repression defect that is highly statistically significant compared to the wild-type ($P < 2e\text{-}16$, Wilcoxon rank-sum test), whereas the *tod6Δ* mutant shows only subtle repression differences compared to the wild-type with marginal significance ($P = 0.01$–0.03, Wilcoxon rank-sum test). (**D**) The distribution of median Dot6-GFP levels in individual cells of indicated strains ($n = 50$ each strain) exposed to 50 μg/mL doxycycline. This dose produces Dot6-GFP protein levels in the *TET-DOT6* strain comparable to *msn2Δmsn4Δ* cells. (**E**) As shown in (**A**) but for wild-type cells paired with TET-inducible *DOT6* both grown side-by-side in 50 μg/mL doxycycline before and after NaCl treatment. (**F**) Box-plot of data from (**E**) for 379 genes. All four *TET-DOT6* samples show weaker repression compared to their paired wild-type sample ($P < 1e\text{-}7$, Wilcoxon rank-sum test). Boxplots show median (line) and 0.25 and 0.75 quartiles (box), with whiskers extending from minimum to maximum excluding outliers (circles) that are <0.25 quartile–1.5× interquartile range or >0.75 quartile–1.5× interquartile range. Exact *P* values provided in Dataset EV1. Source data are available online for this figure.

 © The Author(s)

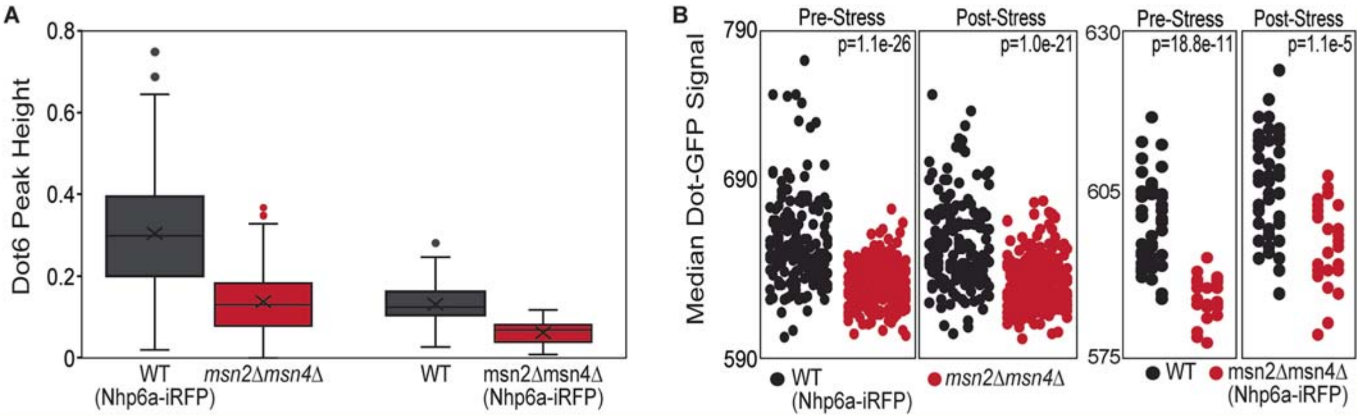

Figure EV4. Msn2/4 effects on Dot6 activity are not an artifact of iRFP.

One consideration was if iRFP expressed in one strain affected GFP signal within the same strain. To ensure that our results in Fig. 4 were not due to unanticipated effects of iRFP, we generated a new set of strains in which the *msn2Δmsn4Δ* cells, rather than the wild-type, carried the distinguishing iRFP signal. We found that the trends discussed in the main text were not affected by which strain carried the iRFP marker. (**A**) Distribution of Dot6 acute stress peak height across wild-type and *msn2Δmsn4Δ* cells when the wild-type carried expressed Nhp6a-iRFP (left, WT $n = 173$, *msn2Δmsn4Δ* $n = 270$) or when the *msn2Δmsn4Δ* strain expressed Nhp6a-iRFP (right, WT $n = 49$, *msn2Δmsn4Δ* $n = 29$). Despite some differences in signal for experiments done with different laser power, *msn2Δmsn4Δ* cells showed weaker Dot6-GFP nuclear translocation signal in both sets of experiments ($P = 8.8e\text{-}11$, left, $P = 1.1e\text{-}5$, right, Wilcoxon rank-sum test). (**B**) Distribution of median Dot6-GFP signal within the cells, scored before (0–72 min) or after (120–216 min) NaCl treatment for wild-type cells and *msn2Δmsn4Δ* cells as described in (**A**). p, Wilcoxon rank-sum test. Boxplots show median (line) and 0.25 and 0.75 quartiles (box), with whiskers extending from minimum to maximum excluding outliers (circles) that are <0.25 quartile–1.5× interquartile range or >0.75 quartile–1.5× interquartile range. Source data are available online for this figure.

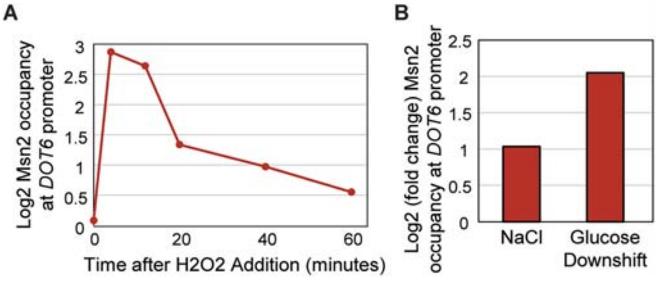

Figure EV5. Msn2 binds *DOT6* promoter under various stresses.

(**A**) Log_2 enrichment of Msn2 occupancy relative to the whole-cell extract at the *DOT6* promoter in response to 0.4 mM H_2O_2 (from Huebert et al, 2012). (**B**) Log_2 (fold change) of Msn2 occupancy at the *DOT6* promoter (ranging from 0 to −1000 bp from Ni et al and +/− 250 bp surrounding the Msn2 STRE element in the *DOT6* promoter) in response to 30 min of 0.6 M NaCl (left, from Ni et al, 2009) or 20 min after shift from glucose to glycerol (right, from Elfving et al, 2014) compared to the corresponding measurement in unstressed cells.

© The Author(s)
